# Psychometric evaluation of the near activity visual questionnaire presbyopia (NAVQ-P) and additional patient-reported outcome items

**DOI:** 10.1186/s41687-024-00717-9

**Published:** 2024-04-09

**Authors:** Joel Sims, Brigitte Sloesen, Sarah Bentley, Christel Naujoks, Rob Arbuckle, Sima Chiva-Razavi, Ben Pascoe, Jan Stochl, Amy Findley, Paul O’Brien, James S. Wolffsohn

**Affiliations:** 1 Adelphi Values, Patient-Centered Outcomes, Bollington, UK; 2 Novartis Pharma AG, Basel, Switzerland; 3 School of Optometry, College of Health and Life Sciences, Aston University, Birmingham, UK; 4 Charles University, Prague, Czechia

**Keywords:** Presbyopia, Patient-reported outcome, Clinical outcome assessment, Psychometric validation, Near vision function

## Abstract

**Background:**

The Near Visual Acuity Questionnaire Presbyopia (NAVQ-P) is a patient-reported outcome (PRO) measure that was developed in a phakic presbyopia population to assess near vision function impacts. The study refined and explored the psychometric properties and score interpretability of the NAVQ-P and additional PRO items assessing near vision correction independence (NVCI), near vision satisfaction (NVS), and near vision correction preference (NVCP).

**Methods:**

This was a psychometric validation study conducted using PRO data collected as part of a Phase IIb clinical trial (CUN8R44 A2202) consisting of 235 randomized adults with presbyopia from the US, Japan, Australia, and Canada. Data collected at baseline, week 2, and months 1, 2, and 3 during the 3-month trial treatment period were included in the analyses to assess item (question) properties, NAVQ-P dimensionality and scoring, reliability, validity, and score interpretation.

**Results:**

Item responses were distributed across the full response scale for most NAVQ-P and additional PRO items. Confirmatory factor analysis supported the pre-defined unidimensional structure and calculation of a NAVQ-P total score as a measure of near vision function. Item deletion informed by item response distributions, dimensionality analyses, item response theory, and previous qualitative findings, including clinical input, supported retention of 14 NAVQ-P items. The 14-item NAVQ-P total score had excellent internal consistency (α = 0.979) and high test-retest reliability (Intraclass Correlation Coefficients > = 0.898). There was good evidence of construct-related validity for all PROs supported by strong correlations with concurrent measures. Excellent results for known-groups validity and ability to detect change analyses were also demonstrated. Anchor-based and distribution-based methods supported interpretation of scores through generation of group-level and within-individual estimates of meaningful change thresholds. A meaningful within-patient change in the range of 8-15-point improvement on the NAVQ-P total score (score range 0–42) was recommended, including a more specific responder definition of 10-point improvement.

**Conclusions:**

The NAVQ-P, NVCI, and NVS are valid and reliable instruments which have the ability to detect change over time. Findings strongly support the use of these measures as outcome assessments in clinical/research studies and in clinical practice in the presbyopia population.

**Supplementary Information:**

The online version contains supplementary material available at 10.1186/s41687-024-00717-9.

## Background

Presbyopia occurs when the physiologically normal age-related reduction in the eye’s focusing range reaches a point, when optimally corrected for distance vision, where the clarity of near vision is insufficient to satisfy an individual’s requirements [1, 2].

Several patient-reported outcome (PRO) instruments have been developed to assess patient-reported near-vision function, however few have been validated as disease specific measures to support efficacy endpoints in presbyopia clinical trials [3–8]. An initial instrument review identified the Near Activity Visual Questionnaire (NAVQ) as a potentially suitable instrument to measure near vision function [8]. However, the items did not reflect changes in technology that have occurred since the questionnaire was developed (e.g., the increase in digital technology use), and the measure was not validated in a purely phakic presbyopia population [9, 10]. There are a number of considerations when managing pseudo-phakic presbyopia in comparison to phakic presbyopia such as navigating corneal scars and residual corneal irregularities from prior incisions, increased prevalence of some symptoms such as dry eyes [9] and differences in visual function such as poorer intermediate vision [11, 12]. As a result, research was conducted to modify the NAVQ for it to reflect current use of digital screens and to confirm its content validity in people with phakic presbyopia [3, 4, 8], in line with best practice guidelines for PRO development [2–8, 13–16]. The research was conducted in two phases: phase one; qualitative research to modify and assess the content validity of the NAVQ-P and phase two; psychometric validation of the NAVQ-P. Phase one included an initial critical review of the NAVQ instrument content and revision to items, a social media listening study (to explore the lived experience of presbyopia) and three rounds of combined concept elicitation (CE) and cognitive debriefing (CD) interviews with an international sample of healthcare professionals (HCPs) and individuals with phakic presbyopia to gather evidence on the content and face validity of the updated NAVQ– the NAVQ-P [2–4]. Additional single-item instruments for the assessment of near vision correction independence (NVCI), near vision correction preference (NVCP), and near vision satisfaction (NVS) were also developed to support efficacy endpoints, along with two global items to assess patient global impression of severity of near vision function (PGI-S) and patient global impression of change in near vision function (PGI-C). The additional instruments were developed in parallel to the NAVQ-P and were subject to the same rigorous development and assessment process [2].

The purpose of this study (phase two) was to establish evidence of the psychometric properties and score interpretability of the NAVQ-P and additional instruments. To achieve this aim, the NAVQ-P, NVCI, NVCP, and NVS were included in a Phase IIb trial and analysis was conducted to support consideration of item reduction and finalise scoring, to evaluate the psychometric properties of the resulting scores, and to provide estimates of meaningful change thresholds that could be considered clinically relevant.

## Methods

### Study design

The psychometric analyses presented in this study were conducted using data collected from a Phase IIb dose-ranging study to evaluate the safety and efficacy of UNR844 in participants with presbyopia, a randomized, placebo-controlled, double-masked, multiple-arm, parallel-group, multi-center study (ClinicalTrials.gov identifier: NCT04806503). The 13-month study consisted of a one-week run-in period, a three-month treatment course with the study treatment (UNR844) and/or placebo and a nine-month treatment holiday period. Participants were randomized equally to one of five treatment arms dosed with various concentrations of UNR844 in both eyes for three months.

Participants completed the NAVQ-P, NVCI, NVS, NVCP, PGI-S, and PGI-C instruments at Baseline, Week 2, and Months 1, 2 and 3 during the three-month treatment period using an electronic PRO (ePRO) device. Distance-corrected near visual acuity (DCNVA) was also assessed at each of these timepoints. These assessments were also administered monthly during the treatment-free follow-up period at Month 4 through 12, but data from those timepoints was not included in the psychometric evaluation analyses (Supplementary File 1). All analyses used data pooled across treatment arms.

### Participant sample and recruitment

A total sample of 225 presbyopia participants were targeted for the Phase IIb study. Participants were recruited from 20 centers in the United States (US), Australia, Canada, and Japan. Participants were required to provide written informed consent, be a phakic male or female participant aged 45 to 55 years inclusive at the Screening visit, have a monocular and binocular DCNVA at 40 cm distance worse than 0.3 logMAR at the Screening and Baseline visits, and binocular DCNVA at Baseline could not differ by more than 0.1 logMAR from the corresponding assessment at the Screening visit. Full eligibility criteria can be found in Supplementary File 2.

Purposive sampling was used to ensure good representation across demographic characteristics including age, region and disease condition.

### Overview of instruments

#### Near activities visual Questionnaire-Presbyopia (NAVQ-P)

The initial version of the NAVQ-P consisted of 15 items which assess near vision functioning in individuals [10]. A recall period of ‘the past seven days’ was specified for all items. The items were scored on a four-point verbal descriptor Likert scale, ranging from ‘no difficulty’ (0) to ‘extreme difficulty’ (3). An additional N/A response ‘I did not do this activity in the past seven days’ was also included. Throughout the NAVQ-P, a higher score indicates greater impairment to near vision. The conceptual framework for the 15-item NAVQ-P can be found in Supplementary File 3.

As detailed in the development paper for the original NAVQ, a single total score was calculated by summing each of the responses with median imputation for N/A responses [10]. Items have been removed/added and item wording has been revised since that version [2, 4], and following the analyses described in this paper, the scoring has since been revised. In line with the objectives of this study, an updated scoring algorithm was developed following item reduction and dimensionality analyses.

#### Near vision correction independence (NVCI)

The Near Vision Correction Independence (NVCI) instrument is a single item designed to assess dependency on near vision correction methods. The response scale assesses the amount of time that vision correction is needed and ranges from ‘none of the time’ (0) to ‘all of the time’ (4). A higher score indicates a greater level of dependence on near vision correction methods. A recall period of ‘the past seven days’ is specified.

#### Near vision satisfaction (NVS) instrument

The Near Vision Satisfaction (NVS) instrument is a single item designed to assess satisfaction with near vision. The response scale ranges from ‘very dissatisfied’ (0) to ‘very satisfied’ (4), with a higher score indicating greater near vision satisfaction. A recall period of ‘the past seven days’ is specified.

#### Near vision correction preference (NVCP) instrument

A single item Near Vision Correction Preference (NVCP) instrument was administered with the NAVQ-P and additional instruments. The instrument asks which method of vision correction the respondent prefers with response options for the study treatment, reading glasses, contact lenses, a magnifier glass, and ‘no preference’. No recall period is specified for this instrument.

Supplementary measures were administered concurrently during the study and were used to support the psychometric validation analysis. These included the patient global impression of severity (PGI-S) and change (PGI-C) items, which are single items with categorical response options designed to capture the patient’s perception of overall presbyopia severity (PGI-S) and change in overall presbyopia severity (PGI-C) at the time of completion. In addition to this, Distance Corrected Near Visual Acuity (DCNVA) was assessed, measured binocularly using an electronic visual acuity system, provided as logMAR scores. These supplementary measures were administered to support psychometric analyses and are also referred to as anchor measures.

### Statistical and data analysis methods

Analysis populations are detailed in Table 1. Key analyses performed on the NAVQ-P and additional instruments to evaluate their psychometric properties are outlined in Table 2. Analysis was conducted in four stages corresponding to the assessment of item properties (Stage 1), dimensionality and scoring (Stage 2), score reliability and validity (Stage 3), and interpretation of scores (Stage 4). Analyses were conducted using SAS® version 9.4 or higher [17], R Version 3.6.0 or above and Mplus Version 8. Item Response Theory (IRT) and Confirmatory Factor Analysis (CFA) were used concurrently to compare and contrast results.

**Table 1:** Analysis populations

Analysis population	Description
Randomized population	All randomized participants as defined by the trial protocol
Cross-sectional analysis population	Participants who have completed the NVCI item, NVS, and all items of the NAVQ-P at Month 2. This population was used for all cross-sectional analyses performed at Month 2. Month 2 was selected since it was expected to have the greatest distribution of scores across the sample.
Test-retest analysis population	The test-retest analysis population consisted of participants who were determined as stable between Months 2 and 3 (primary test-retest analysis population), or, stable between Week 2 and Month 1 timepoints (secondary test-retest analysis population). Stability between these timepoints was defined using two different criteria: 1. No change in PGI-S 2. < 0.14 logMAR change in DCNVA Test-retest reliability analysis was conducted separately for each of these populations and stability definitions. The threshold for DCNVA logMAR change was informed by previous literature [28, 29].
Interpretability analysis populations	For interpretability analyses, ‘stable’, ’improved’, and ‘worsened’ groupings were defined from the randomised population using multiple anchors including the PGI-S, PGI-C, and DCNVA. All anchor-based analyses were performed examining changes between baseline and Months 1, 2, and 3, with the change to Month 3 considered the primary analysis. The anchor groups defining change were as follows: **PGI-S (1 grade)**: •Improved ( > = 1-point PGI-S improvement) •Stable (0-point PGI-S change) •Worsened ( > = 1-point PGI-S worsening) **PGI-S (2 grade)**: •Improved ( > = 2-point PGI-S improvement) •Stable (< 2-point PGI-S change) •Worsened ( > = 2-point PGI-S worsening) **PGI-C**: •Improved (A little better/Much better) •Stable (No change) •Worsened (A little worse/Much worse) **DCNVA**: •Improved ( > = 0.14 logMAR decrease) •Stable (< 0.14 absolute logMAR change) •Worsened ( > = 0.14 logMAR increase)

**Table 2:** Overview of statistical analysis methods

Analysis	Description
**Stage 1: Item properties**	
Quality of completion	• The quality of completion for the NAVQ-P, NVCI, and NVS was assessed at the item level in the randomized population (*N* = 235) at Baseline, Week 2, Month 1, Month 2, and Month 3. For the NAVQ-P, form level (whole PRO) missing data was also evaluated following finalisation of scoring.
Item response distributions and floor and ceiling effects	• Item response distributions for the NAVQ-P items, NVCI, NVS and NVCP at Baseline and Month 3 were examined to identify any skewed distributions or overly preferred response options for a given item.
**Stage 2: Dimensionality and scoring**	
Inter-item correlations	• Inter-item correlations provided an initial exploration of dimensionality and were examined using polychoric correlation coefficients between each pair of items in the NAVQ-P in the cross-sectional analysis population at Month 2. This was done to ensure each item measured a distinct concept without any redundancy. Items that correlated highly with one another (> 0.90) or correlated < 0.40 were flagged for review.
Internal consistency reliability	• Internal consistency reliability, concerned with the homogeneity of items belonging to the same domain, were evaluated using Cronbach’s alpha coefficient (≥ 0.70 for good internal consistency) [30]. • The impact of item removal on internal consistency reliability was examined. Cronbach’s alpha was calculated with each item removed from their respective scores to assess the impact. • Internal consistency was assessed at Month 2 in the cross-sectional analysis population for the NAVQ-P.
Confirmatory factor analysis (CFA)	• Confirmatory factor analysis (CFA) of the NAVQ-P was conducted using data from the cross-sectional analysis population at Month 2 to assess the dimensionality of the 15-item NAVQ-P to inform item deletion and overall scoring. • Factor analytic models employed a weighted least square mean and variance adjusted (WLSMV) estimator, with theta parametrisation. • Model fit indices were used to assess model fit (CFI = Comparative Fit Index; TLI = Tucker Lewis Index; RMSEA = Root Mean Square Error of Approximation and SRMR = Standardizes Root Mean Square Residual). • Model fit indices were evaluated against the following desirable thresholds with the intended use to guide model fit assessment and not as strict cut-offs (CFI > 0.95, TLI > 0.95, RMSEA < 0.08 and SRMR < 0.05). • Deciding between a weighted or unweighted summary score was informed through comparison of constrained (where factor loadings are constrained to be equal) vs. unconstrained (factor loadings are freely estimated) CFA models. • In the case that factor loadings can be considered equal (i.e. model with constrained factor loadings will not fit significantly worse compared to model with freely estimated factor loadings) across items, an unweighted sum score was proposed [18]. • The NVCI, NVS, and NVCP were not included in these analyses as they are measuring distinct concepts that are not directly related to near vision functioning and were not expected to form part of the NAVQ-P score. Relationships of the single-item measures were instead assessed within the convergent validity analysis.
IRT analyses of NAVQ-P	• The NAVQ-P was assessed through item response theory (IRT) analyses to inform item properties, dimensionality, and scoring. The analysis was performed for the cross-sectional analysis population at Month 2 to assess whether the NAVQ-P was unidimensional. • The Rating Scale Model (RSM) was applied, with the N/A response treated as missing for this analysis. • Item characteristic curves were used to assess probability of responses and weak or overlapping item response categories. • Person fit was evaluated through assessment of standardized fit residuals and number/proportion of participants with fit residuals outside of the range 0 ± 2.5 were summarized. • Local dependency was assessed by Yens Q3 statistic with any residual correlation greater than the average residual correlation + 0.30 highlighting potential redundancy and interdependence [19, 20]. • Person separation reliability was assessed which is comparable to Cronbach’s alpha coefficient, values > 0.70 are deemed acceptable. • Item fit was assessed by the infit mean square (MNSQ) and outfit MNSQ to highlight observed responses that deviate from the Rasch model expectation. Values between 0.5–1.5 indicate acceptable item fit and are productive for measurement. • Item person maps were employed to flag overlapping items and any gaps in item location on the latent trait continuum.
Item reduction for the NAVQ-P	• Item reduction was considered for the NAVQ-P based on the analyses of item properties and dimensionality, but also considering previous qualitative findings and the clinical relevance and importance of the items. • IRT and internal consistency analyses were repeated iteratively following the deletion of items until a final item set was decided upon.
**Stage 3: Reliability and validity of scores**	
Reliability	
Scale-level test-retest reliability	• The stability of scale-level scores between Months 2 and 3, and Week 2 and Month 1 was assessed in the primary and secondary test-retest analysis populations respectively using PGI-S and DCNVA-defined stable groups. • Intraclass correlation coefficient (ICC) was calculated for continuous scores. The following cut-offs were employed to interpret ICC values: values < 0.40 were considered indicative of poor reliability, values between 0.40–0.75 indicated fair to good reliability, values > 0.75 indicated excellent reliability [31]. • The stability of NVCI and NVS scores was assessed by calculating weighted Kappa coefficients interpreted as follows: ≥0.75 excellent; 0.40- <0.75 as fair; <0.40 as poor [31].
Construct-related validity	
Convergent validity	• Convergent validity was evaluated by calculating correlations of the DCNVA with the NAVQ-P, NVCI, and NVS using data collected in the cross-sectional analysis population at Month 2. • Scores assessing similar or related concepts were expected to have strong correlations (*r* ≥ 0.5) thereby demonstrating convergent validity.
Known-groups analysis	• Construct validity was also assessed using the known-groups method, to evaluate differences in mean PRO scores between groups of participants who differ in severity as defined by PGI-S and DCNVA scores. • Known-group comparisons were assessed using Month 2 data in the cross-sectional analysis population.
Ability to detect change over time	• Ability to detect change over time analyses focused on the evaluation of changes in PRO scores over time to demonstrate that observed improvements (or reductions) in those scores correspond to improvements (or worsening) in external criteria (anchors) also related to the construct. • Ability to detect change was assessed using data from Baseline, Months 1, 2, and 3, with change from Baseline to Month 3 considered the primary analysis. • The following pre-specified cut-offs were used to interpret the magnitude of each effect size (ES): small (ES = 0.20), moderate (ES = 0.50), and large (ES = 0.80) [32].
**Stage 4: Interpretation of scores**	
Anchor-based methods	• Anchor-based methods were used to identify participants who experienced an important change in their condition, by exploring the association between changes on the NAVQ-P, NVCI, and NVS and the anchor measures (PGI-S, PGI-C, and DCNVA). • All anchor-based analyses were performed in the interpretability analysis population by examining changes between Baseline and Months 1, 2, and 3, with the change to Month 3 considered as the primary analysis. • A theoretical justification between the anchor and target instrument should exist and should be empirically demonstrated [14, 33, 34]. The suitability of proposed anchors was tested using a polyserial correlation coefficient or Spearman’s rank to establish the relationship between the change in the anchor and change in each PRO score between Baseline and Month 3. Anchors with correlations of < 0.30 were not taken forward for analysis. • Each anchor deemed to have a sufficient relationship with the PRO scores was used to define groups of participants who experienced improvement, no change or worsening according to the interpretability analysis populations. • The mean change in PRO score was calculated for participants classified as improved, stable, and worsened (meaningful within-group change). The meaningful between-group difference for each anchor was defined as the difference in mean change PRO score between the improved and stable groups. • Receiver operating characteristics (ROC) curve analysis was used to find the change in PRO score that optimally discriminates between improved and stable groups defined by the anchors. • Empirical Cumulative Distribution Functions (eCDFs) and Probability density functions (PDFs) were also plotted to aid comparison of different possible responder definitions on the PRO scores [13]. • Tables showing change from Baseline to Month 3 in NAVQ-P, NVCI, and NVS in terms of various percentiles, by baseline PGI-S, were also developed to explore any baseline dependency of meaningful change.
Distribution-based methods	• Distributional properties of the NAVQ-P, NVCI, and NVS scores were used to guide potential responder definitions estimated from anchor-based approaches, identifying the amount of change that exceeds measurement error [16, 35]. • These included 0.5 of the standard deviation (SD) at Baseline and the standard error of measurement (SEM).
Triangulation	• Triangulation was conducted by consolidating the different meaningful change estimates derived from anchor-based and distribution-based methods to support identification of an appropriate range of meaningful change values [24–26]. • Correlation-weighted average estimates of meaningful change from the anchor-based methods were also used to converge on a range of potential meaningful change estimates [27].

## Results

### Sample characteristics

The randomized population consisted of 235 participants in total. Of those, 227 individuals with presbyopia completed the NVCI, NVS, and all items of the NAVQ-P at Month 2 (cross-sectional analysis population). Demographic and clinical characteristics for the cross-sectional analysis population are summarised in Table 3. The mean age of this sample was 50.9 years and consisted of slightly more females than males and mostly participants from the US. The majority were white and non-Hispanic or non-Latino. Most participants were of a mild severity of presbyopia, as assessed by a DCNVA score better than 0.6 logMAR (i.e., better than 20/80 Snellen equivalent). Most participants also required a vision correction aid and were not myopic, defined as any refractive error based on manifest refraction that is <-0.75 spherical equivalent in at least one eye.

**Table 3:** Demographic and clinical characteristics of the cross-sectional analysis population at baseline (*N* = 227)

Sample characteristics	Statistic or N (%)
Age (years)	
N	227
Mean (SD)	50.9 (2.76)
Median	51.0
Min, Max	45, 55
Sex	
Female	132 (58.1%)
Male	95 (41.9%)
Country	
US	131 (57.7%)
Japan	45 (19.8%)
Australia	35 (15.4%)
Canada	16 (7.0%)
Ethnicity	
Not Hispanic or Latino	207 (91.2%)
Hispanic or Latino	18 (7.9%)
Not reported	2 (0.8%)
Race*	
White	160 (70.5%)
Asian	51 (22.5%)
Black or African American	14 (6.2%)
Multiple	2 (0.9%)
Does the participant need vision correction aid?	
Yes	155 (68.3%)
No	72 (31.7%)
DCNVA	
DCNVA better than 0.6 logMAR (i.e., better than 20/80 Snellen equivalent)	191 (84.1%)
DCNVA of 0.6 logMAR or worse (i.e., 20/80 or worse Snellen equivalent)	34 (15.0%)
Missing	2 (0.9%)
Is participant myopic?	
Yes	32 (14.1%)
No	195 (85.9%)

SD: Standard Deviation; DCNVA: Distance-Corrected Near Visual Acuity; Cross-sectional analysis population includes participants who have completed the NVCI item, NVS and all items of the NAVQ-P at Month 2

### Item properties

Due to administration of the instruments via ePRO without the option to skip items, missing data was minimal and less than 5% of randomised participants (ranging from 0.4% at baseline to 4.3% at Month 3) did not complete the NAVQ-P or other instruments at any given timepoint.

For the NAVQ-P, NVCI, and NVS, responses for all items were relatively evenly distributed across the full response scale across timepoints with a higher proportion endorsing the more severe response options, though this was not considered of concern (Supplementary File 4 and Supplementary File 5).

### Dimensionality and scoring

#### NAVQ-P inter-item correlations and factor analysis

**Fig. 1 Path diagram of 15-item NAVQ-P confirmatory factor analysis.**
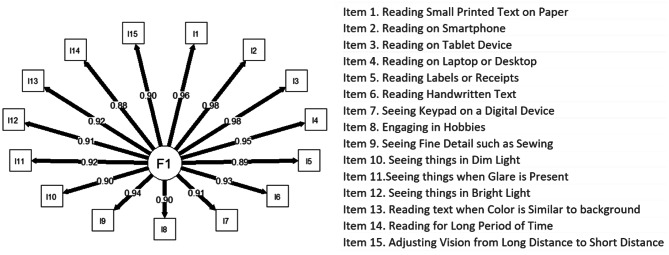
Values associated with arrows represent the factor loadings of each item on the single factor (F1); I = Item. NAVQ-P item concepts shown in legend. Analysis was conducted using responses to NAVQ-P items in the cross-sectional analysis population at Month 2

Inter-item correlations ranged between 0.716 and 0.983, suggesting that all items are assessing closely related concepts, however a few correlations were above 0.90 which may suggest potential item redundancy (see Supplementary File 6). Confirmatory factor analysis (CFA) was conducted to assess the hypothesized unidimensional structure of the NAVQ-P. Results from the unconstrained CFA supported the *a priori* unidimensional structure showing good model fit (Table 4) and similar factor loadings across all items (Fig. 1). Comparison of the unconstrained model with the constrained CFA model showed the constrained model was only marginally worse (with respect to RMSEA and SRMR) and still well-fitting the observed data. This provides support for a unidimensional construct and unweighted NAVQ-P summary score [18]. Interestingly, CFI and TLI showed worse fit for the unconstrained model (Table 4).

**Table 4:** Unconstrained and Constrained Model fit Indices for the 15-item NAVQ-P

Model fit index	Unconstrained Model	Constrained model
CFI	0.966	0.991
TLI	0.966	0.991
RMSEA	0.095	0.137
SRMR	0.029	0.049
AIC	4552	4609
BIC	4758	4767

CFI = Comparative Fit Index; TLI = Tucker Lewis Index; RMSEA = Root Mean Square Error of Approximation; SRMR = Standardizes Root Mean Square Residual; AIC = Akaike Information Criterion; BIC = Bayesian Information Criterion. A lower AIC and/or BIC value indicates a better model fit

#### NAVQ-P item response theory (IRT) performance

Infit and outfit statistics identified items with observed responses that deviated from the Rasch model expectations, almost all NAVQ-P items indicated acceptable infit and outfit statistics (range 0.539–1.353). Only Item 1 (Reading small, printed text on paper) and Item 9 (Seeing fine detail, such as sewing) had outfit and/or infit values < 0.50; these values are slightly outside of the prespecified acceptable range (< 0.50 or > 1.50), but were not so low that they would degrade the measurement. Therefore, no items were flagged for removal based on this analysis. Person fit was evaluated through examination of standardized fit residuals which ranged from − 5.16 to 4.91, with 24 residual values outside of the prespecified (0 ± 2.5) range (*n* = 24/226; 10.6%), a small percentage which is unlikely to impact practically on NAVQ-P measurement.

Item characteristic curves (item parameters are reported in Supplementary File 7) illustrated that response options reflected the appropriate level of near vison functioning severity observed in the participants (e.g., participants with more severe symptoms would select the more extreme response), with no unexpected or overlapping response options. These findings are supportive of the adequacy of the response scale (see Supplementary File 8 for item characteristic curves).

**Fig. 2:**
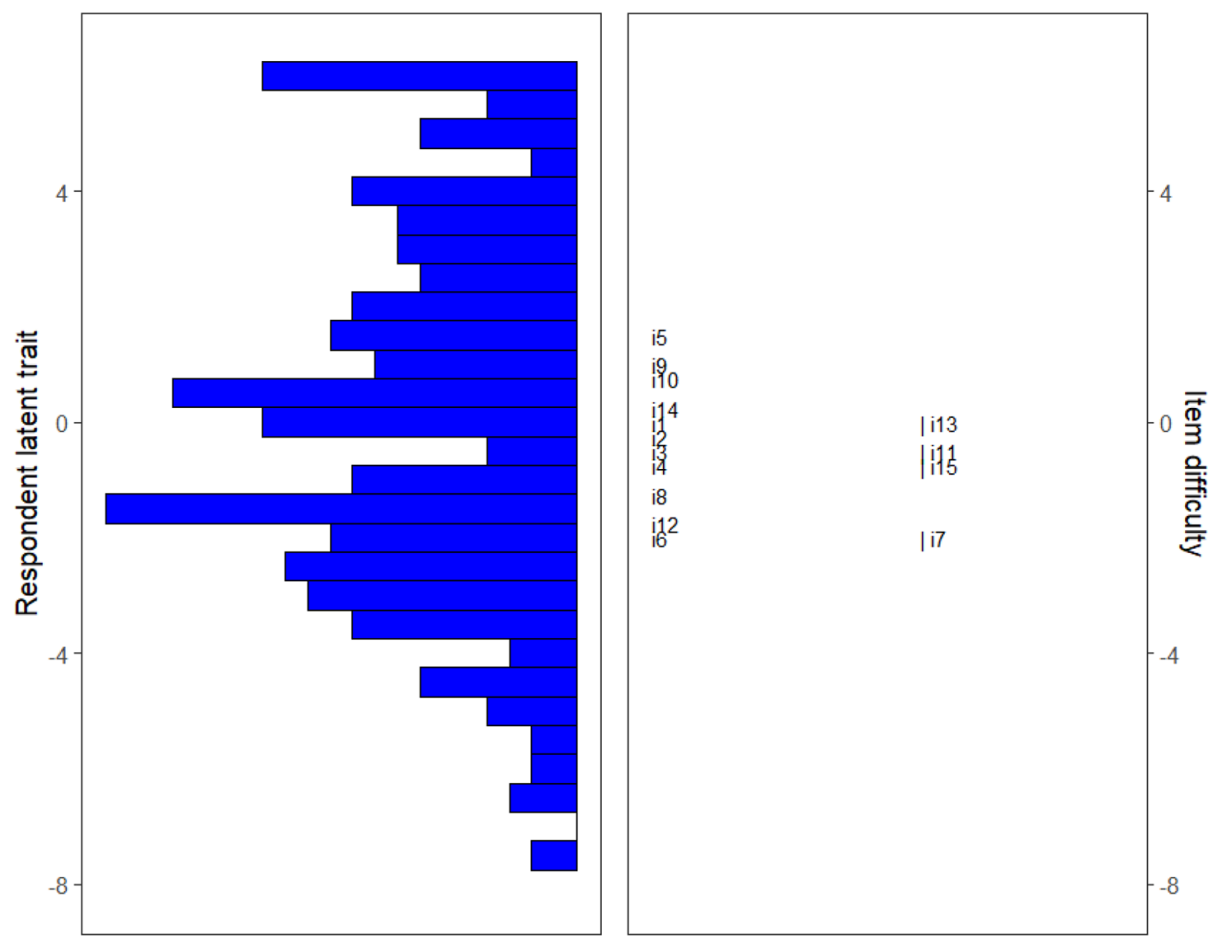
Item-person map representing participants and NAVQ-P items on the same latent trait in the cross-sectional analysis population at Month 2 i1 to i15 (i = Item) indicate location of NAVQ-P item in respect to respondent latent trait/item difficulty scale.

Item-person maps were generated to illustrate the location of participants in the sample along the latent trait continuum, alongside the difficulty of endorsement for each of the NAVQ-P items. Item difficulties of all 15 NAVQ-P items are located within − 2 to + 2 units of the logit scale with a relatively good spread of items across differing levels of presbyopia severity. Some items overlap on the item difficulty logit scale such as Item 3 (Reading on a tablet device) and Item 11 (Seeing things when glare is present) among others (Fig. 2). Although these items are close on the difficulty parameter scale, this provides higher measurement precision, and each item assesses slightly different aspects of near vision functioning/presbyopia so are considered of value to retain from a content validity perspective. Figure 2 also suggests Item 5 (Reading labels or receipts) was most likely to be endorsed by individuals with more severe near vision functioning impairment (higher NAVQ-P scores), also supported by having the highest mean response score (M = 2.21). In contrast, Item 6 (Reading handwritten text) and Item 7 (Seeing keypad on a digital device) were most likely to be endorsed by participants with less severe near vision functioning impairment (lower NAVQ-P scores)– supported by both items having the lowest mean response score (M = 1.41).

To assess local dependency/redundancy of the NAVQ-P items, Yen’s Q3 statistic was produced to assess residual correlations between item pairs [19, 20]. The highest residual correlations for item pairs involved Item 3. Residual correlations exceeding the cut-off of 0.234 are displayed in Supplementary File 9. The person separation index [21] for the NAVQ-P was 0.965, indicating the collection of items can efficiently separate the participants that are being measured, and that the sample is of adequate size and composition to locate the items on the latent trait.

#### NAVQ-P item reduction

Following consideration of results from stage 1 and 2 psychometric analyses, the study team discussed finalisation of NAVQ-P scoring and potential item deletion, including input from an expert optometrist in presbyopia (JSW; an author of this paper and developer of the NAVQ). Only Item 3 (Reading on a tablet device) was deleted resulting in a 14-item NAVQ-P instrument. It was judged that all other items were of value to retain to assess a range of near vision functioning concepts. Items discussed for removal are detailed in Table 5 with justification for deletion/retention.

**Table 5:** Rationale for possible items for deletion

Item	Rationale			Decision
	Inter-item correlation	Item properties	IRT modelling	
3. Reading on Tablet Device	Potential redundancy with item 2 (0.973) and 4 (0.983) Item 2. Reading on a smartphone Item 4. Reading on a laptop or desktop	Most participants noted doing this activity between Baseline and Month 3	Potential local dependency with Item 2 and 4	**Remove item**
8. Engaging in Hobbies	Inter-item correlations were less than 0.9	High percentage of participants endorsing the N/A response option (20.0-25.1%) between Baseline and Month 3	No evidence of local dependency	**Retain**– despite 20% of participants endorsing N/A option, the item assesses a slightly different concept to other items
14. Reading for Long Period of Time	Inter item correlations were less than 0.9	Most participants noted doing this activity between Baseline and Month 3	Potential local dependency with Item 15 (Adjusting vision from long distance to short distance)	**Retain**– despite potential local dependency with item 15, the items assess slightly different concepts

#### Finalisation of NAVQ-P scoring

Stage 2 analyses provided evidence of a unidimensional factor structure supporting calculation of one NAVQ-P summary score. For the 14-item NAVQ-P, it was decided to treat ‘N/A’ responses as equivalent to missing data and to impute ‘N/A’ responses and missing data using the median of the items that were responded to by that participant. The decision was also taken to apply the half scale rule for calculating the 14-item NAVQ-P summary score (i.e., if ≥ 50% of responses to the NAVQ-P are ‘N/A’ or missing, do not calculate a score for that respondent) [22]. The total sum of all item scores (following median imputation and application of half-scale rule) appropriately represents the summary score for the 14-item NAVQ-P, and is referred to as the NAVQ-P total score.

Form-level missing data was subsequently evaluated to assess missingness of the NAVQ-P total score in the randomised population. Form-level missing data was minimal (ranging from 3% of the sample with missing NAVQ-P total score at baseline to 8% at Month 3) which was not considerably different to item-level missing data.

### Reliability and validity of scores

#### Internal consistency reliability

The Cronbach’s alpha coefficient for the 14-item NAVQ-P was very high (α = 0.979). When the Cronbach’s alpha was calculated with each item deleted, the Cronbach’s alpha value did not increase above the overall 14-item Cronbach’s alpha value (Supplementary File 10). Results provide support for retaining all 14 items. Of note, the Cronbach’s alpha value for the 15-item NAVQ-P with Item 3 retained was α = 0.981, therefore only negligibly different to the 14-item Cronbach’s alpha indicating that deletion of Item 3 was not detrimental to the internal consistency reliability of the NAVQ-P total score.

#### Scale-level test-retest reliability

Excellent test-retest reliability was observed for the NAVQ-P total score in both the primary and secondary test-retest analysis populations. All lower bounds of 95% confidence intervals ICC values were 0.866 or larger, thus demonstrating strong agreement/reproducibility of NAVQ-P total scores within the 2 and 4-week intervals analysed among stable participants (Supplementary File 11).

Weighted Kappa coefficients indicated fair test-retest reliability for the NVS (0.487–0.655) and fair to moderate for the NVCI (0.642–0.753) across the different stability definitions, showing some evidence of agreement of scores, with better agreement observed within the 4-week interval from Month 2 to Month 3 that was later in the trial, by which time participants’ presbyopia might have been expected to be more stable.

#### Construct-related validity

##### Convergent validity

Although in the expected direction, the correlation between the NAVQ-P total score and the DCNVA did not reach the hypothesized moderate or high-level correlation of ≥ 0.50 and exhibited a low correlation (*r* = 0.220). However, a high correlation of *r* = 0.770 with the PGI-S score was observed supporting convergent validity with a participant-reported measure of severity (Table 6).

**Table 6:** Convergent validity of NAVQ-P total score and NVCI and NVS scores with DCNVA and PGI-S scores at Month 2

	Cross-sectional Analysis Population (*N* = 227)			
	DCNVA		PGI-S	
Target Score	n	Correlation Coefficient	n	Correlation Coefficient
NAVQ-P Total Score	222	0.220	223	0.770
NVCI Score	226	0.198	227	0.734
NVS Score	226	-0.266	227	-0.676

Cross-sectional analysis population includes participants who have completed the NVCI item, NVS and all items of the NAVQ-P at Month 2; n = Number of participants used to calculate Spearman correlation coefficient (values for both scores); Values represent Spearman correlation coefficients calculated between the PRO target score, DCNVA (logMAR scores) and PGI-S scores

These patterns of results were also observed for the NVCI and NVS scores, with low correlations observed with DCNVA, but strong correlations with the PGI-S in the expected direction supporting convergent validity (Table 6).

##### Known-groups validity

For the NAVQ-P, there were statistically significant differences between the three PGI-S defined severity groups (*F*_*2,220*_=146.88, *p* < 0.001), with monotonically increasing mean NAVQ-P total scores in accordance with greater PGI-S severity. There were also large between-group effect sizes for the Moderate and Severe/Very severe PGI-S groups in respect to the None/Mild reference group. Statistically significant pairwise differences were exhibited for all comparisons of the mean NAVQ-P total score between PGI-S groups. There were no statistically significant differences in NAVQ-P total score between known-groups defined by the DCNVA anchor. However, the sample size for the more severe DCNVA group ( > = 0.6 logMAR, *n* = 15) was smaller than the sample size for the less severe group (< 0.6 logMAR, *n* = 207; Table 7).

**Table 7:** Known-groups validity of NAVQ-P Total Score and NVCI and NVS scores at Month 2 using PGI-S and DCNVA-defined severity groups

	Cross-sectional analysis population (*N* = 227)			
Target Score / Known-groups	n	Mean (SD)	Between-Groups Effect Size	Pair-wise comparison p-value
NAVQ-P Total Score				
PGI-S*				
None/Mild (reference)	67	13.16 (8.25)		
Moderate	80	23.85 (8.25)	1.30	<0.001
Severe/Very severe	76	35.68 (7.04)	2.95	<0.001
**DCNVA**				
DCNVA < 0.6 logMAR (better visual acuity)	207	24.48 (11.91)		
DCNVA > = 0.6logMAR (worse visual acuity)	15	26.47 (12.80)	0.17	0.536
NVCI				
PGI-S*				
None/Mild (reference)	69	1.59 (1.02)		
Moderate	81	2.84 (0.83)	1.35	< 0.001
Severe/Very severe	77	3.68 (0.68)	2.43	< 0.001
**DCNVA**				
DCNVA < 0.6 logMAR (better visual acuity)	210	2.75 (1.18)		0.681
DCNVA > = 0.6 logMAR (worse visual acuity)	16	2.63 (1.36)	-0.11	
NVS				
PGI-S*				
None/Mild (reference)	69	2.20 (0.88)		
Moderate	81	1.23 (0.69)	-1.23	< 0.001
Severe/Very severe	77	0.51 (0.79)	-2.03	< 0.001
**DCNVA**				
DCNVA < 0.6 logMAR (better visual acuity)	210	1.32 (1.05)		0.099
DCNVA > = 0.6 logMAR (worse visual acuity)	16	0.88 (0.72)	-0.43	

*For the PGI-S, participants responding with ‘None’ or ‘Mild’ were grouped together as were participants responding with ‘Severe’ or ‘Very severe’ in order to ensure adequate sample size in each PGI-S group. The between groups effect size is using Hedge’s g compared to the reference group (ref). Hedge’s g is calculated as the difference in means ((comparison group) - (reference group)) divided by the pooled standard deviation. P-value based on a two-sample t-test testing mean score differences between groups. NAVQ-P total scores range from 0–42, with higher scores indicating greater symptom severity. NVCI and NVS scores range from − 4 to + 4. A higher score on the NVS indicates greater satisfaction with near vision, without a near vision correction aid. A higher score on the NVCI indicates greater dependency on a near vision correction aid

The NVCI and NVS showed similar results for the known-groups analysis as for the NAVQ-P with statistically significant differences between the PGI-S defined severity groups (*F*_*2,224*_= 110.8 and 84.6 respectively, *p* < 0.001), statistically significant pairwise differences for all PGI-S group comparisons, and large between-group effect sizes. As with the NAVQ-P, known-groups comparisons of NVS or NVCI scores using DCNVA did not show any significant differences between groups (Table 7).

Based on the known-groups comparison for the PGI-S groups, there is support for the known-groups validity of the NAVQ-P total score as well as NVCI and NVS scores, in respect to participant-reported severity.

##### Ability to detect change

Participants were grouped according to the pre-defined anchor groups of ‘improved’, ‘no change’, and ‘worsened’ (Table 1).

**Fig. 3:**
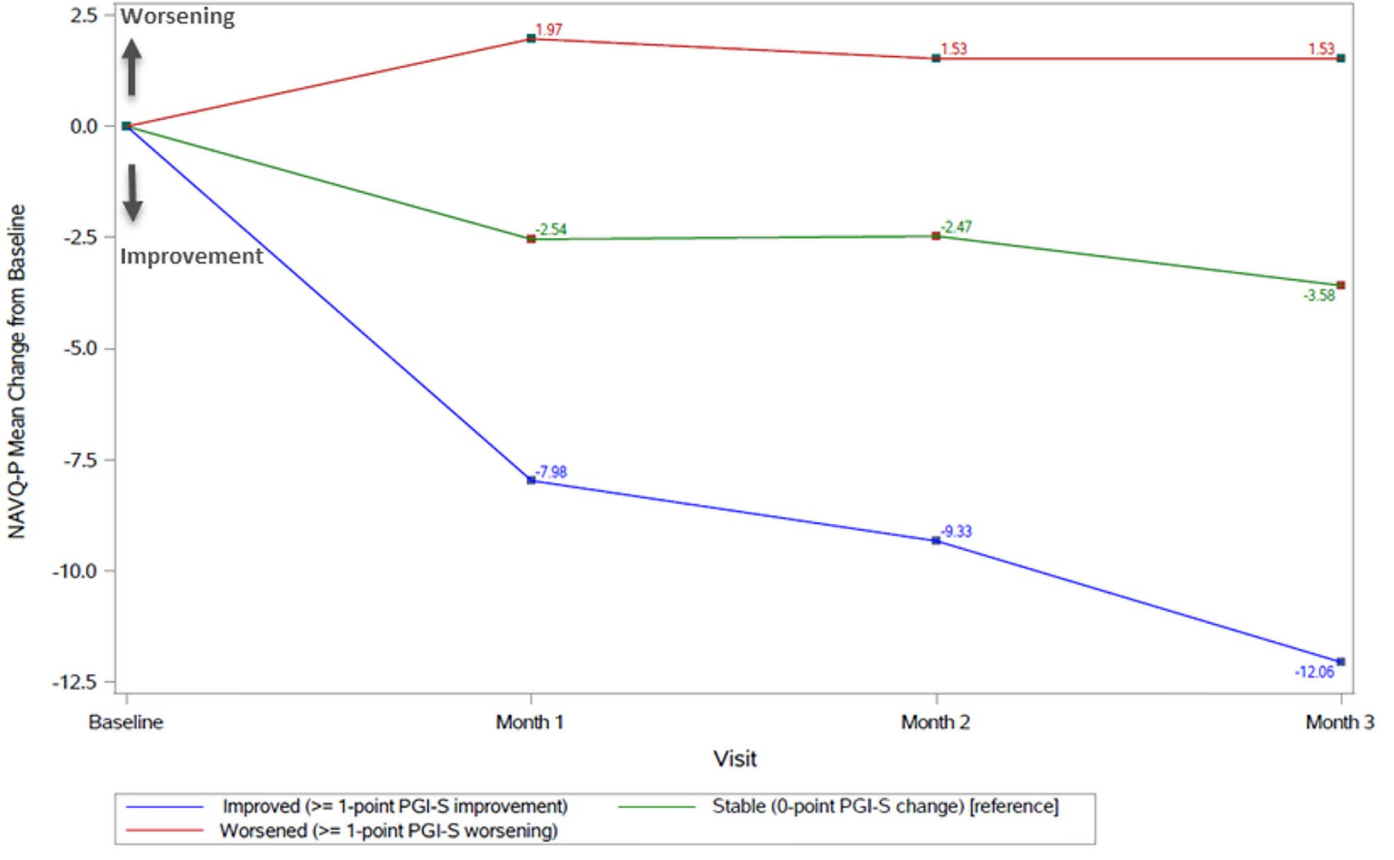
NAVQ-P total score mean change from baseline at Month 1, 2, and 3 according to change in PGI-S (PGI-S 1-grade anchor)

The NAVQ-P total score was able to detect improvement between Baseline and Month 3 as defined by the PGI-S, PGI-C, and DCNVA anchors, with large effect sizes (ES -1.27 to -0.96) for change in the NAVQ-P total score observed in the improved group across all anchors used. Within-group change in the improved group was statistically significant across all anchors (*p* < 0.001). There was a small effect size (ES = 0.17) for the PGI-S worsened group, but in the expected direction, although not statistically significant (*p* = 0.145). The change in the worsened group for the PGI-C and DCNVA anchors could not be appropriately interpreted due to their small sample size. The effect sizes for the stable participants were small (ES range: -0.19 to -0.50) and consistently smaller than the improved group as expected. Results of a one-way ANOVA showed statistically significant differences in mean change between anchor groups (*p* ≤ 0.001). The results for changes from Baseline to Month 1 and Month 2 were consistent with the results at Month 3 (Fig. 3). Similar results were seen for the NVCI and NVS (full results for ability to detect change analysis can be found in Supplementary File 12).

#### Interpretation of scores

Estimates of meaningful change were triangulated from multiple anchor-based analyses with distribution-based estimates to converge on a range of potential thresholds for meaningful individual- and group-level change for the NAVQ-P total score and NVCI and NVS scores.

#### Anchor-based methods

Change in the PGI-S and responses to the PGI-C correlated well with the change in NAVQ-P total score as well as change in NVCI and NVS. Change in DCNVA correlated poorly with change in each of these target scores, therefore the DCNVA anchor was not taken forward to support anchor-based interpretation of scores (Table 8).

**Table 8:** Correlations between change in NAVQ-P total score, NVCI, and NVS and change in proposed anchors between Baseline and Month 3

Target Score	Spearman correlation coefficient		
	PGI-S	PGI-C	DCNVA (logMAR)
NAVQ-P total score	0.618	-0.549	0.247
NVCI	0.472	-0.547	0.245
NVS	-0.498	-0.583	-0.285

Values represent Spearman’s rank correlation coefficients for change from Baseline to Month 3

##### Group-level change

All group-level meaningful change estimates for meaningful within-group change and meaningful between-group difference are provided in Supplementary File 13. Change from Baseline to Month 3 results have been included in the triangulation for group-level estimates of meaningful change (Table 9).

**Table 9:** ROC-based responder definitions for NAVQ-P total score at Month 3

Anchor	AUC (95% CI)	ROC curve estimates (Change from Baseline to Month 3)			
		Method 1	Method 2	Method 3	Method 4
NAVQ-P Total Score					
PGI-S (1 grade)	0.776 (0.706,0.845)	-8.0	-6.0	-8.0	-8.0
PGI-S (2 grade)	0.892 (0.832,0.953)	-8.0	-11.0	-8.0	-14.0
PGI-C	0.831 (0.775,0.887)	-8.0	-5.0	-8.0	-6.0
NVCI					
PGI-S (1 grade)	0.695 (0.625,0.766)	-1.0	-1.0	-1.0	-1.0
PGI-S (2 grade)	0.761 (0.651,0.871)	-1.0	-1.0	-1.0	-1.0
PGI-C	0.773 (0.714,0.831)	-1.0	-1.0	-1.0	-1.0
NVS					
PGI-S (1 grade)	0.711 (0.640,0.782)	1.0	1.0	1.0	1.0
PGI-S (2 grade)	0.828 (0.740,0.916)	2.0	2.0	2.0	2.0
PGI-C	0.811 (0.757,0.865)	1.0	1.0	1.0	1.0

AUC: Area Under the Curve; CI: Confidence Interval; ROC: Receiver Operating Characteristic. Responder thresholds are based on the change score that optimally discriminates between improved and stable groups defined by the anchor.

AUC and 95% CI are calculated from the ROC curve for each score using the randomised population.

Method 1: Threshold defined by maximising the sum of sensitivity and specificity, also referred to as Youden’s J index.

Method 2: Threshold defined by minimising the absolute difference between sensitivity and specificity.

Method 3: Threshold defined by minimising the sum of 1- sensitivity and 1– specificity

Method 4: Threshold defined by selecting the point in the ROC space which minimises the sum of squares.

Negative NAVQ-P and NVCI change scores and positive NVS change scores represent improvement in symptoms.

##### Individual-level change

Results of the Receiver Operating Characteristic (ROC) curve analysis showed strong predictive ability of the NAVQ-P total score, NVCI, and NVS scores to discriminate between stable and improved groups of participants shown by large AUC estimates across all anchors. Most ROC estimates suggest 8.0-point improvement on the NAVQ-P total score (range 5-14-point improvement) and 1-point improvement on the NVCI and NVS to be the optimal thresholds for discriminating improved and stable participants (Table 9).

**Table 10:** Correlation-weighted average estimates of meaningful improvement for NAVQ-P total score, NVCI, and NVS using change from baseline to Month 3

Meaningful change	Anchor	Estimates	Correlation-weighted estimate	Recommended thresholds
NAVQ-P Total Score				
Between-group difference	PGI-S (1 grade)	-8.50	11.2-point change	11.2-point improvement
	PGI-S (2 grade)	-14.80		
	PGI-C	-10.20		
Within-group change	PGI-S (1 grade)	-12.10	14.8-point change	14.8-point improvement
	PGI-S (2 grade)	-19.80		
	PGI-C	-12.00		
Within-patient change (responder definition)*	PGI-S (1 grade)	-8.0	9.5-point change (ROC only) 12.1-point change (ROC and within-group change)	Range of 8 to 15-point improvement (10-point change recommended as proposed threshold)
	PGI-S (2 grade)	-14.0		
	PGI-C	-8.0		
NVCI				
Between-group difference	PGI-S (1 grade)	-0.6	0.9-point change	0.9-point improvement
	PGI-S (2 grade)	-1.1		
	PGI-C	-1.0		
Within-group change	PGI-S (1 grade)	-0.8	1.0-point change	1.0-point improvement
	PGI-S (2 grade)	-1.3		
	PGI-C	-0.9		
Within-patient change (responder definition)*	PGI-S (1 grade)	-1.0	1.0-point change (ROC only) 1.0-point change (ROC and within-group change)	1.0-point improvement
NVS				
Between-group difference	PGI-S (1 grade)	0.8	1.1-point change	1.1-point improvement
	PGI-S (2 grade)	1.4		
	PGI-C	1.2		
Within-group change	PGI-S (1 grade)	1.3	1.6-point change	1.6-point improvement
	PGI-S (2 grade)	2.0		
	PGI-C	1.4		
Within-patient change (responder definition)*	PGI-S (1 grade)	1.0	1.3-point change (ROC only) 1.4-point change (ROC and within-group change)	1.0-point improvement
	PGI-S (2 grade)	2.0		
	PGI-C	1.0		

*Only ROC estimates using Method 4 (minimising sum of squares) were included in the correlation-weighted estimate of meaningful within-patient change since this method is proven mathematically to be closest to the top-left corner of the ROC curve. Correlation-weighted average estimates of meaningful within-patient change (responder definition) were calculated using the ROC curve-based estimates and separately with both the ROC-based and within-group change estimates included.

**Fig. 4:**
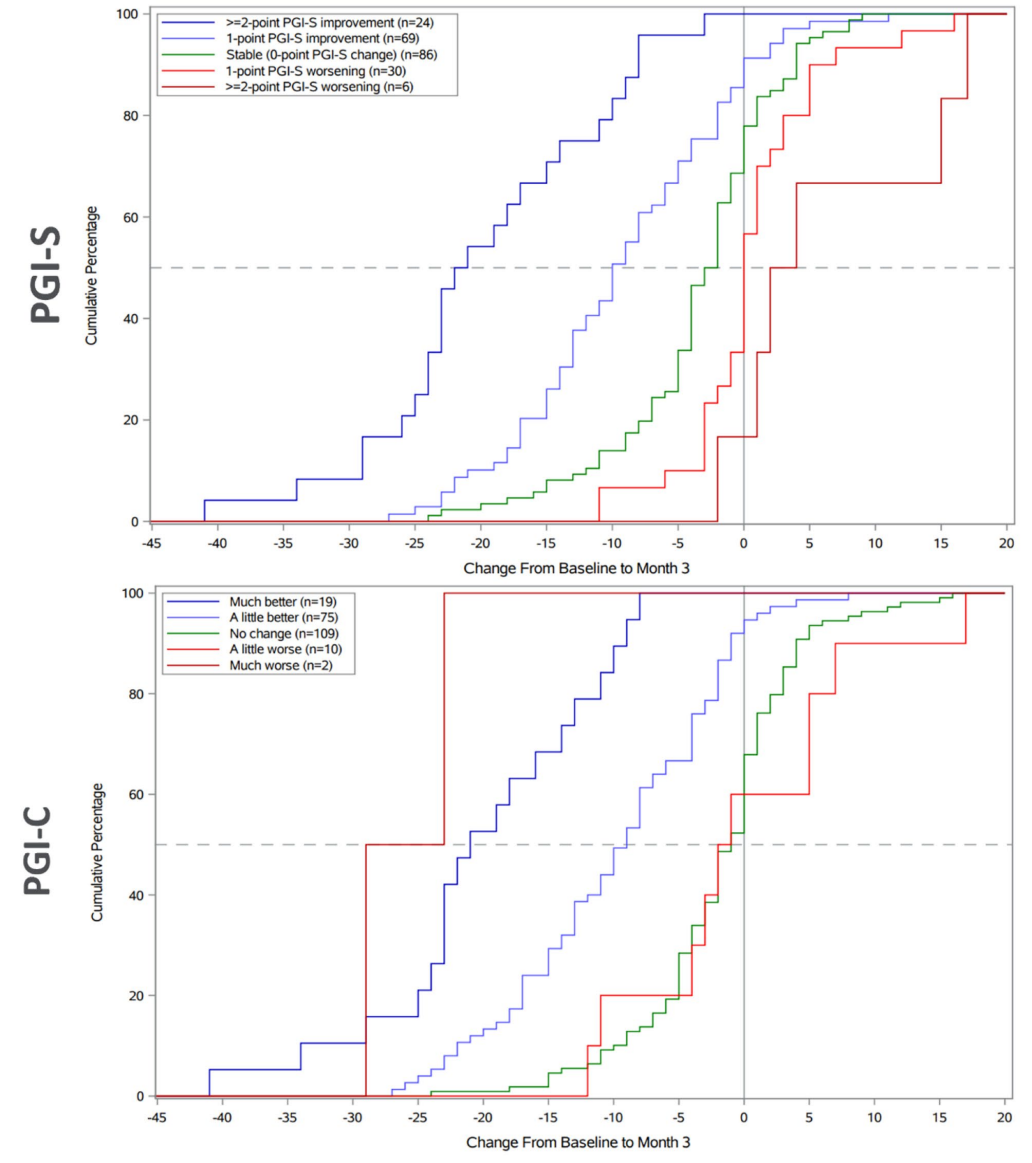
eCDF plot of NAVQ-P total score change from Baseline to Month 3 by PGI-S (top) and PGI-C (bottom) anchor groups

Responder thresholds from the anchor-based ROC curve analysis were used and group-level change estimates to aid identification of a possible range of responder thresholds based on examination of eCDF and PDF plots. For the NAVQ-P total score, the range of thresholds identified from the ROC curve-based analysis appeared compatible with the distribution of change observed across the PGI-S and PGI-C anchor groups, with a low proportion of stable participants who would be considered ‘responders’ for any value in the initial proposed range (5-14-point improvement), and high proportions of improved participants who would be considered responders (see Fig. 4 for eCDF plot). This was also observed for change from baseline in the NVCI and NVS. These results are further discussed as part of the triangulation of estimates (which also take into account the distribution-based estimates).

#### Distribution-based methods

Half standard deviation (0.5 SD) and standard error of measurement (SEM) using baseline scores were calculated to explore measurement variability and guide potential responder definitions for the NAVQ-P total score. Given a 0.5 SD value of 4.842 and SEM of 1.936, a within-individual change of 5-points on the NAVQ-P total score may be considered as a guide, indicating the level of change that exceeds measurement error. A within-patient change of 1-point improvement on the NVCI and NVS may be considered the threshold for exceeding measurement error since possible individual-level score changes on these items are limited to whole values (Supplementary File 14).

#### Triangulation of meaningful change estimates

Table 10 reports the correlation-weighted average estimates for the anchor-based group-level estimates and individual-level estimates of meaningful change on the NAVQ-P total score, NVCI, and NVS (correlations were reported in Table 10). A within-patient change in the range of 8-15-point improvement on the NAVQ-P total score appears to be appropriate since any value in this range appears to identify mainly improved participants and a very small proportion of stable participants (based on examination of the eCDF plots; see Fig. 4), and exceeds the distribution-based estimate of ≥ 5-point change. A more specific responder definition of 10-point improvement is recommended based on this range (Table 10). Meaningful change thresholds are recommended for the NVCI and NVS using the same approach (Table 10).

## Discussion

Psychometric evaluation of the NAVQ-P and additional PRO instruments has been conducted in line with regulatory best-practice guidelines for development of PRO instruments, adding to the previous qualitative evidence supporting the content validity of the NAVQ-P in phakic presbyopia [2, 3].

The sample of participants in this study had a range of demographic and clinical characteristics and is considered representative of the phakic presbyopia population. Notably, participants were aged 45–55 years given the clinical trial criteria, therefore results are only representative of this age group and not older individuals with presbyopia. However, this is the critical age group whose near ability is decreasing and choices needed to be made on presbyopia amelioration options. The sample consisted of participants recruited from four countries (United States, Japan, Australia, and Canada) of various races, providing some level of confidence in the generalizability of the results. However, to further enhance the generalisability of the results from this study, future research would ideally include a more culturally diverse sample of participants including a broader range of countries beyond those included in this study and would also be conducted in a ‘real-world’ sample, rather than a clinical trial sample.

Overall results from this study provide good evidence supporting the psychometric validity of the NAVQ-P and established the dimensionality and scoring of the instrument. Consistent with the high inter-item correlations, further examination of the dimensionality of the NAVQ-P provided strong support for a unidimensional instrument. Item response distributions, inter-item correlations, Rasch analysis, and previous qualitative interviews [2], including input from clinical experts, informed the removal of Item 3 (Reading on a tablet device) from the NAVQ-P, resulting in a valid and reliable 14-item instrument. Specifically, qualitative interviews highlighted that reading on a tablet device was less relevant in phakic presbyopia with only *n* = 15/35 reporting the concept as relevant to their experience [2]. It was judged valuable to retain all other items such that the measure provides credible evidence of the impact of presbyopia on all important aspects of near vision functioning. Nevertheless, the high inter-item correlations and high internal consistency results suggest that, if there was a preference for a shorter version of the measure in the future, such a measure could likely still be highly valid, reliable, and sensitive to change. However, previous qualitative interviews highlighted that certain activities may not be completed regularly, therefore the 14-item version would likely better assess different aspects of the patient experience in a seven-day period [2]. While there was a relatively good spread of items to assess differing levels of presbyopia severity, the NAVQ-P could arguably benefit from items that discriminate at the more severe end of near vision functioning. However, in the context of correcting near vision in presbyopia, it is typically of more interest to be able to differentiate at the middle and lower end of near vision functioning severity with sensitivity to differentiate between mild/moderate presbyopia, as demonstrated by the NAVQ-P.

The psychometric properties of the 14-item NAVQ-P total score showed excellent internal consistency reliability, test-retest reliability, and good evidence of construct-related validity. As part of the assessment of convergent validity (which is an aspect of construct-related validity), weak correlations between the NAVQ-P total score and DCNVA (logMAR scores) and for change from baseline in these scores were observed. Similarly, the known-groups analysis which used DCNVA to define groups did not find significant results. However, these results are in line with previous research which has shown the NAVQ-P to demonstrate only relatively weak correlations with near visual acuity measures similar to the DCNVA (*r* = 0.32) [10]. DCNVA is assessed by the detection of high contrast, capital letters at a fixed working distance rather than functional vision [23], which shows the benefit of the NAVQ-P in better capturing the individual’s perception of “near vision functioning”. As the DCNVA is a direct measure of visual acuity, it could be argued that it is not surprising there is not a strong relationship with the NAVQ-P which measures visual function. As a large correlation (*r* = 0.770) between the NAVQ-P total score and PGI-S was observed, and statistically significant differences in the known-groups defined using the PGI-S, there was clear support for the construct validity of the NAVQ-P to capture patient-reported severity of near vision functioning in presbyopia. However, it is a limitation of the study that the strongest convergent validity and known-groups validity evidence is based on groups defined by the PGI-S items, which were developed as anchors specifically for use in this study. Further study of the discriminative ability of the NAVQ-P that uses other independently established measures of near vision functioning is warranted.

Importantly, evidence in support of ability to detect change over time was observed for the NAVQ-P even when change groups were defined using DCNVA, providing good evidence that the NAVQ-P is sensitive to changes over time. All of these findings are consistent with the psychometric results for the earlier version of the instrument (the NAVQ), which were equally compelling [10]. Examining correlations with other external and ideally validated measures of near visual functioning or near visual acuity would be useful to provide additional evidence of construct-related validity beyond the patient-reported anchors in this study; this aspect of construct validity evaluation was relatively limited in this study.

Triangulation of meaningful change estimates from multiple methods and anchors strengthened the proposed recommendations for what constitutes a meaningful change at the group-level and individual-level, aligning with recommended best practice from the literature and current regulatory guidance [14, 16, 24–27]. For estimates of meaningful within-patient change on the NAVQ-P total score, a range of possible thresholds were generated (8-15-point improvement) from across the different anchors. Future studies using the NAVQ-P for the assessment of near vision functioning in presbyopia can use these thresholds for defining meaningful change to aid interpretation of changes in scores. It must be acknowledged that the data for these analyses was collected from a trial that was unsuccessful and did not find significant differences between treatment groups. Thus, the ability to detect change and meaningful change results should be interpreted in that context. This limitation is mitigated by the fact that all analyses were performed using data pooled across treatment groups, and consideration that the change groups were defined using anchors. Nevertheless, it is possible that further study of the NAVQ-P in a trial that includes a successful intervention may yield higher meaningful change estimates or point towards the upper end of the range suggested above being more appropriate. Further study is warranted.

Strong psychometric properties and similar patterns of results were also seen for the NVCI and NVS as observed for the NAVQ-P total score with score interpretation thresholds provided. However, in contrast to the NAVQ-P, the NVCI and NVS demonstrated only fair to moderate test-retest reliability. The lower test-retest reliability for these instruments relative to the NAVQ-P may be due to the definition of stability used to define the test-retest population. While these participants may be stable with regard to near vision functioning based on the PGI-S and PGI-C anchors, these anchors may not be closely related to the concepts assessed by the NVCI and NVS which may be partly influenced by factors other than near vision functioning.

## Conclusion

The findings reported from this study provide evidence that the NAVQ-P provides a measure of patient-reported near vision functioning in presbyopia that is valid, reliable, and has ability to detect change over time. Similarly, the NVCI, NVS, and NVCP instruments demonstrated strong psychometric properties as measures of participant satisfaction with treatment, dependence on visual aids, and vision correction aid preference, respectively, however further examination of their reliability over time within a more suitably defined stable population may be useful to provide stronger evidence of test-retest reliability. Recommendations for interpreting changes in these scores have been provided.

The findings strongly support the adequacy of these measures as ‘fit-for-purpose’ instruments for inclusion as assessments to support endpoints in future clinical studies in the presbyopia population or for use in clinical practice to assess changes in these concepts over time.

### Electronic supplementary material

Below is the link to the electronic supplementary material.

Supplementary Material 1

Supplementary Material 2

Supplementary Material 3

Supplementary Material 4

Supplementary Material 5

Supplementary Material 6

Supplementary Material 7

Supplementary Material 8

Supplementary Material 9

Supplementary Material 10

Supplementary Material 11

Supplementary Material 12

Supplementary Material 13

Supplementary Material 14

Supplementary Material 15

Supplementary Material 16

## Data Availability

The data that support the findings of this study are available from Novartis Pharma AG but restrictions apply to the availability of these data, which were used under license for the current study, and so are not publicly available. Data are however available from the authors upon reasonable request and with the permission of Christel Naujoks.

## Abbreviations

BIC: Bayesian information criterion
CFA: Confirmatory factor analysis
CFI: Comparative fit index
DCNVA: Distance-corrected near visual acuity
eCDFs: Empirical cumulative distribution functions
ePRO: An electronic PRO device
ES: Effect size
NAVQ-P: Near visual acuity questionnaire-presbyopia
NVCI: Near vision correction independence
NVCP: Near vision correction preference
NVS: Near vision satisfaction
PDF: Probability density function
PGI-C: Patient global impression of change in near vision function
PGI-S: Patient global impression of severity of near vision function
PRO: patient-reported outcome
RMSEA: Root mean square error of approximation
ROC: Receiver operating characteristics
RSM: Rating scale model
SD: Standard deviation
SEM: Standard error of measurement
SRMR: Standardized root mean square residual
TLI: Tucker lewis index
WLSMV: Weighted least square mean and variance adjusted estimator
