# Supplementary material for: Psychometric evaluation of the near activity visual questionnaire presbyopia (NAVQ-P) and additional patient-reported outcome items
Source: J Patient Rep Outcomes. 2024 Apr 9;8:41. doi: 10.1186/s41687-024-00717-9 (PMC11004101; doi:10.1186/s41687-024-00717-9)
Supplement: Supplementary file 1 — Supplementary Material 1 [file 41687_2024_717_MOESM1_ESM.docx]

| **Schedule of assessments relating to patient-reported outcome and visual acuity measures to support psychometric validation** | | | | | | |
| --- | --- | --- | --- | --- | --- | --- |
| Assessment | Treatment | | | | | Post-Treatment Monthly Follow-up* |
| Visit Name | BL | Week 2 | Month 1 | Month 2 | Month 3 | Month 4-Month 12 |
| Patient-reported outcome assessments | | | | | | |
| Near Activity Visual Questionnaire Presbyopia (NAVQ-P) | X | X | X | X | X | X |
| Near Vision Satisfaction (NVS) instrument | X | X | X | X | X | X |
| Near Vision Correction Independence (NVCI) instrument | X | X | X | X | X | X |
| Near Vision Correction Preference (NVCP) instrument |  | X | X | X | X | X |
| Other measures | | | | | | |
| Patient Global Impression of Severity (PGI-S) item |  | X | X | X | X | X |
| Patient Global Impression of Change (PGI-C) item |  | X | X | X | X | X |
| Distance-corrected near visual acuity (DCNVA) | X | X | X | X | X | X |
| BL = Baseline. * Data from the post-treatment follow-up (treatment holiday) period was not included in any of the psychometric analyses. Note a 1-week run-in screening period prior to the baseline assessment was included in the study design but was not included in any of the analyses reported in this psychometric evaluation study. | | | | | | |
