# Supplementary material for: Psychometric evaluation of the near activity visual questionnaire presbyopia (NAVQ-P) and additional patient-reported outcome items
Source: J Patient Rep Outcomes. 2024 Apr 9;8:41. doi: 10.1186/s41687-024-00717-9 (PMC11004101; doi:10.1186/s41687-024-00717-9)
Supplement: Supplementary file 2 — Supplementary Material 2 [file 41687_2024_717_MOESM2_ESM.rtf]

	Cross-sectional Analysis Population (N=227)	
	Cronbach's alpha
All NAVQ-P items	Cronbach's alpha
Item 3 removed	
	
Overall Cronbach's alpha	0.981 (n=222)	0.979 (n=223)	
	
Cronbach's alpha if item deleted	
  Item 1. Reading Small Printed Text	0.979	0.977	
  Item 2. Reading Smartphone	0.979	0.977	
  Item 3. Reading Tablet Device	0.979		
  Item 4. Reading Laptop or Desktop	0.979	0.977	
  Item 5. Reading Labels on Receipts	0.980	0.978	
  Item 6. Reading Handwritten Text	0.980	0.977	
  Item 7. Keypad on a Smartphone	0.980	0.978	
  Item 8. Engaging in Your Hobbies	0.980	0.977	
  Item 9. Seeing Closer Objects	0.979	0.977	
  Item 10. Seeing Things in Dim Light	0.980	0.978	
  Item 11. Glare is Present	0.979	0.977	
  Item 12. Reading Outdoors in Daylight	0.980	0.978	
  Item 13. Color to the Background	0.979	0.977	
  Item 14. Long Period of Time	0.980	0.978	
  Item 15. Immediately After Looking	0.980	0.978	
	
