# Supplementary material for: Psychometric evaluation of the near activity visual questionnaire presbyopia (NAVQ-P) and additional patient-reported outcome items
Source: J Patient Rep Outcomes. 2024 Apr 9;8:41. doi: 10.1186/s41687-024-00717-9 (PMC11004101; doi:10.1186/s41687-024-00717-9)
Supplement: Supplementary file 3 — Supplementary Material 3 [file 41687_2024_717_MOESM3_ESM.docx]

| Table 7. NAVQ-P total score scale-level test-retest reliability | | | | |
| --- | --- | --- | --- | --- |
|  | **Primary TRTAP (Month 2 - Month 3)** | | **Secondary TRTAP (Week 2 - Month 1)** | |
| **Score / Anchor Stability Definition** | **n** | **ICC/Kappa Estimate (95% CI)** | **n** | **ICC/Kappa Estimate (95% CI)** |
| NAVQ-P Total Score | | | | |
| PGI-S (No change) | 136 | 0.906 (0.866, 0.934) | 133 | 0.926 (0.897, 0.947) |
| DCNVA (<0.14 logMAR change) | 181 | 0.923 (0.895, 0.943) | 183 | 0.898 (0.865, 0.923) |
| NVCI | | | | |
| PGI-S (No change) | 139 | 0.753 (0.682, 0.824) | 138 | 0.685 (0.600, 0.771) |
| DCNVA (<0.14 logMAR change) | 189 | 0.751 (0.687, 0.815) | 192 | 0.642 (0.565, 0.718) |
| NVS | | | | |
| PGI-S (No change) | 139 | 0.653 (0.568, 0.738) | 138 | 0.492 (0.377, 0.607) |
| DCNVA (<0.14 logMAR change) | 189 | 0.655 (0.582, 0.729) | 192 | 0.487 (0.396, 0.578) |
| CI: Confidence Interval; ICC: Intraclass correlation coefficient; calculated using an ICC [2,1] single measurement, absolute agreement, two-way mixed effects model (NAVQ-P total score only); Kappa coefficients represent Weighted Kappa coefficients (NVCI and NVS only). TRTAP: Test-Retest Analysis Population. Primary TRTAP is the primary test-retest analysis population and consists of participants who are determined as stable between Month 2 and Month 3 (4 weeks apart) according to specified anchor definitions. Secondary TRTAP is the secondary test-retest analysis population and consists of participants who are determined as stable between Week 2 and Month 1 (2 weeks apart) according to specified anchor definitions; n: number of participants. | | | | |
