# Supplementary material for: Psychometric evaluation of the near activity visual questionnaire presbyopia (NAVQ-P) and additional patient-reported outcome items
Source: J Patient Rep Outcomes. 2024 Apr 9;8:41. doi: 10.1186/s41687-024-00717-9 (PMC11004101; doi:10.1186/s41687-024-00717-9)
Supplement: Supplementary file 4 — Supplementary Material 4 [file 41687_2024_717_MOESM4_ESM.rtf]

Target Score / Anchor Groups	n	Mean change
(SD)	Median change
(min, max)	Within groups effect size (p-value)	Pair-wise comparisons p-value	One-way ANOVA p-value	
NAVQ-P Total Score	
  Improved (>= 1-point PGI-S improvement)	86	-8.0 (7.95)	-9 (-27, 12)	-0.846 (<.001)	<.001	<.001	
  Stable (0-point PGI-S change) [reference]	98	-2.5 (6.14)	-1 (-24, 14)	-0.254 (<.001)			
  Worsened (>= 1-point PGI-S worsening)	38	2.0 (5.04)	1 (-10, 14)	0.209 (0.021)	<.001		
	

Target Score / Anchor Groups	n	Mean change
(SD)	Median change
(min, max)	Within groups effect size (p-value)	Pair-wise comparisons p-value	One-way ANOVA p-value	
NAVQ-P Total Score	
  Improved (>= 1-point PGI-S improvement)	99	-9.3 (8.81)	-10 (-36, 12)	-1.067 (<.001)	<.001	<.001	
  Stable (0-point PGI-S change) [reference]	85	-2.5 (6.21)	-2 (-20, 11)	-0.251 (<.001)			
  Worsened (>= 1-point PGI-S worsening)	36	1.5 (6.95)	0 (-10, 18)	0.128 (0.196)	0.004		


		

Target Score / Anchor Groups	n	Mean change
(SD)	Median change
(min, max)	Within groups effect size (p-value)	Pair-wise comparisons p-value	One-way ANOVA p-value	
NAVQ-P Total Score	
  Improved (>= 1-point PGI-S improvement)	93	-12.1 (9.39)	-11 (-41, 11)	-1.291 (<.001)	<.001	<.001	
  Stable (0-point PGI-S change) [reference]	86	-3.6 (6.45)	-3 (-24, 9)	-0.377 (<.001)			
  Worsened (>= 1-point PGI-S worsening)	36	1.5 (6.15)	1 (-11, 17)	0.166 (0.145)	<.001		
	

Target Score / Anchor Groups	n	Mean change
(SD)	Median change
(min, max)	Within groups effect size (p-value)	Pair-wise comparisons p-value	One-way ANOVA p-value	
NAVQ-P Total Score	
  Improved (>= 2-point PGI-S improvement)	17	-8.3 (9.37)	-11 (-19, 12)	-0.899 (0.002)	0.068	<.001	
  Stable (<2-point PGI-S change) [reference]	199	-3.8 (7.29)	-2 (-27, 14)	-0.382 (<.001)			
  Worsened (>= 2-point PGI-S worsening)	6	5.2 (5.38)	4 (-1, 13)	0.629 (0.065)	0.009		
	

Target Score / Anchor Groups	n	Mean change
(SD)	Median change
(min, max)	Within groups effect size (p-value)	Pair-wise comparisons p-value	One-way ANOVA p-value	
NAVQ-P Total Score	
  Improved (>= 2-point PGI-S improvement)	29	-13.8 (9.31)	-15 (-36, 5)	-1.536 (<.001)	<.001	<.001	
  Stable (<2-point PGI-S change) [reference]	185	-3.9 (7.59)	-3 (-30, 18)	-0.410 (<.001)			
  Worsened (>= 2-point PGI-S worsening)	6	6.0 (7.16)	3 (-1, 15)	0.363 (0.095)	0.019		
	

Target Score / Anchor Groups	n	Mean change
(SD)	Median change
(min, max)	Within groups effect size (p-value)	Pair-wise comparisons p-value	One-way ANOVA p-value	
NAVQ-P Total Score	
  Improved (>= 2-point PGI-S improvement)	24	-19.8 (9.03)	-22 (-41, -3)	-1.980 (<.001)	<.001	<.001	
  Stable (<2-point PGI-S change) [reference]	185	-5.1 (7.76)	-4 (-27, 16)	-0.539 (<.001)			
  Worsened (>= 2-point PGI-S worsening)	6	6.2 (7.88)	3 (-2, 17)	0.630 (0.114)	0.017		
	

Target Score / Anchor Groups	n	Mean change
(SD)	Median change
(min, max)	Within groups effect size (p-value)	Pair-wise comparisons p-value	One-way ANOVA p-value	
NAVQ-P Total Score	
  4-point PGI-S improvement	0	-	-	-	-	<.001	
  3-point PGI-S improvement	2	-9.5 (13.44)	-10 (-19, 0)	-6.718 (0.500)	0.597		
  2-point PGI-S improvement	15	-8.1 (9.34)	-11 (-19, 12)	-0.868 (0.005)	0.039		
  1-point PGI-S improvement	69	-7.9 (7.63)	-7 (-27, 9)	-0.831 (<.001)	<.001		
  0-point PGI-S change [reference]	98	-2.5 (6.14)	-1 (-24, 14)	-0.254 (<.001)			
  1-point PGI-S worsening	32	1.4 (4.82)	1 (-10, 14)	0.145 (0.117)	<.001		
  2-point PGI-S worsening	5	6.0 (5.57)	5 (-1, 13)	0.653 (0.074)	0.024		
  3-point PGI-S worsening	1	1.0 (-)	1 (1, 1)	-	-		
  4-point PGI-S worsening	0	-	-	-	-		
	

Target Score / Anchor Groups	n	Mean change
(SD)	Median change
(min, max)	Within groups effect size (p-value)	Pair-wise comparisons p-value	One-way ANOVA p-value	
NAVQ-P Total Score	
  4-point PGI-S improvement	0	-	-	-	-	<.001	
  3-point PGI-S improvement	1	-36.0 (-)	-36 (-36, -36)	-	-		
  2-point PGI-S improvement	28	-13.0 (8.43)	-15 (-26, 5)	-1.456 (<.001)	<.001		
  1-point PGI-S improvement	70	-7.5 (7.96)	-8 (-30, 12)	-0.867 (<.001)	<.001		
  0-point PGI-S change [reference]	85	-2.5 (6.21)	-2 (-20, 11)	-0.251 (<.001)			
  1-point PGI-S worsening	30	0.6 (6.67)	0 (-10, 18)	0.065 (0.607)	0.030		
  2-point PGI-S worsening	4	1.5 (2.08)	2 (-1, 4)	0.083 (0.245)	0.019		
  3-point PGI-S worsening	2	15.0 (0.00)	15 (15, 15)	0.849 (<.001)	<.001		
  4-point PGI-S worsening	0	-	-	-	-		
	

Target Score / Anchor Groups	n	Mean change
(SD)	Median change
(min, max)	Within groups effect size (p-value)	Pair-wise comparisons p-value	One-way ANOVA p-value	
NAVQ-P Total Score	
  4-point PGI-S improvement	0	-	-	-	-	<.001	
  3-point PGI-S improvement	3	-26.0 (13.45)	-22 (-41, -15)	-3.329 (0.079)	0.101		
  2-point PGI-S improvement	21	-19.0 (8.32)	-21 (-34, -3)	-1.879 (<.001)	<.001		
  1-point PGI-S improvement	69	-9.4 (7.93)	-10 (-27, 11)	-1.025 (<.001)	<.001		
  0-point PGI-S change [reference]	86	-3.6 (6.45)	-3 (-24, 9)	-0.377 (<.001)			
  1-point PGI-S worsening	30	0.6 (5.44)	0 (-11, 16)	0.070 (0.551)	0.001		
  2-point PGI-S worsening	5	4.4 (7.37)	2 (-2, 17)	0.403 (0.253)	0.071		
  3-point PGI-S worsening	1	15.0 (-)	15 (15, 15)	-	-		
  4-point PGI-S worsening	0	-	-	-	-		
	

Target Score / Anchor Groups	n	Mean change
(SD)	Median change
(min, max)	Within groups effect size (p-value)	Pair-wise comparisons p-value	One-way ANOVA p-value	
NAVQ-P Total Score	
  Improved (A little better/Much better)	78	-9.0 (7.20)	-10 (-25, 9)	-1.064 (<.001)	<.001	<.001	
  Stable (No change) [reference]	133	-0.8 (5.75)	0 (-24, 14)	-0.076 (0.112)			
  Worsened (A little worse/Much worse)	11	-4.5 (10.88)	0 (-27, 10)	-0.507 (0.204)	0.295		
	

Target Score / Anchor Groups	n	Mean change
(SD)	Median change
(min, max)	Within groups effect size (p-value)	Pair-wise comparisons p-value	One-way ANOVA p-value	
NAVQ-P Total Score	
  Improved (A little better/Much better)	90	-9.2 (8.22)	-10 (-30, 7)	-1.020 (<.001)	<.001	<.001	
  Stable (No change) [reference]	118	-1.7 (6.92)	-1 (-20, 15)	-0.170 (0.008)			
  Worsened (A little worse/Much worse)	11	-3.5 (13.84)	0 (-36, 18)	-0.296 (0.427)	0.689		
	

Target Score / Anchor Groups	n	Mean change
(SD)	Median change
(min, max)	Within groups effect size (p-value)	Pair-wise comparisons p-value	One-way ANOVA p-value	
NAVQ-P Total Score	
  Improved (A little better/Much better)	94	-12.0 (8.97)	-11 (-41, 8)	-1.272 (<.001)	<.001	<.001	
  Stable (No change) [reference]	109	-1.8 (6.24)	-1 (-24, 16)	-0.187 (0.004)			
  Worsened (A little worse/Much worse)	12	-4.3 (12.92)	-3 (-29, 17)	-0.519 (0.279)	0.526		
	

Target Score / Anchor Groups	n	Mean change
(SD)	Median change
(min, max)	Within groups effect size (p-value)	Pair-wise comparisons p-value	One-way ANOVA p-value	
NAVQ-P Total Score	
  Improved (>= 0.14 logMAR decrease)	61	-6.9 (8.17)	-4 (-27, 6)	-0.728 (<.001)	<.001	<.001	
  Stable (<0.14 absolute logMAR change) [reference]	159	-2.7 (7.11)	-1 (-19, 14)	-0.276 (<.001)			
  Worsened (>= 0.14 logMAR increase)	0	-	-	-	-		
	

Target Score / Anchor Groups	n	Mean change
(SD)	Median change
(min, max)	Within groups effect size (p-value)	Pair-wise comparisons p-value	One-way ANOVA p-value	
NAVQ-P Total Score	
  Improved (>= 0.14 logMAR decrease)	75	-8.9 (8.43)	-9 (-36, 7)	-0.942 (<.001)	<.001	<.001	
  Stable (<0.14 absolute logMAR change) [reference]	139	-3.0 (8.06)	-2 (-26, 18)	-0.309 (<.001)			
  Worsened (>= 0.14 logMAR increase)	3	3.7 (6.35)	0 (0, 11)	0.235 (0.423)	0.205		
	

Target Score / Anchor Groups	n	Mean change
(SD)	Median change
(min, max)	Within groups effect size (p-value)	Pair-wise comparisons p-value	One-way ANOVA p-value	
NAVQ-P Total Score	
  Improved (>= 0.14 logMAR decrease)	79	-9.4 (10.28)	-9 (-41, 11)	-0.957 (<.001)	<.001	0.001	
  Stable (<0.14 absolute logMAR change) [reference]	132	-4.6 (8.45)	-4 (-26, 17)	-0.495 (<.001)			
  Worsened (>= 0.14 logMAR increase)	1	-7.0 (-)	-7 (-7, -7)	-	-		
	

Target Score / Anchor Groups	n	Mean change
(SD)	Median change
(min, max)	Within groups effect size (p-value)	Pair-wise comparisons p-value	One-way ANOVA p-value	
Near Vision Satisfaction	
  Improved (>= 1-point PGI-S improvement)	89	1.0 (0.89)	1 (0, 3)	1.166 (<.001)	<.001	<.001	
  Stable (0-point PGI-S change) [reference]	102	0.4 (0.61)	0 (-1, 2)	0.498 (<.001)			
  Worsened (>= 1-point PGI-S worsening)	41	0.2 (0.83)	0 (-1, 3)	0.229 (0.197)	0.209		
	

Target Score / Anchor Groups	n	Mean change
(SD)	Median change
(min, max)	Within groups effect size (p-value)	Pair-wise comparisons p-value	One-way ANOVA p-value	
Near Vision Satisfaction	
  Improved (>= 1-point PGI-S improvement)	100	1.2 (0.94)	1 (-1, 3)	1.579 (<.001)	<.001	<.001	
  Stable (0-point PGI-S change) [reference]	88	0.4 (0.84)	0 (-1, 3)	0.547 (<.001)			
  Worsened (>= 1-point PGI-S worsening)	39	0.0 (0.81)	0 (-2, 2)	0.028 (0.844)	0.014		
	

Target Score / Anchor Groups	n	Mean change
(SD)	Median change
(min, max)	Within groups effect size (p-value)	Pair-wise comparisons p-value	One-way ANOVA p-value	
Near Vision Satisfaction	
  Improved (>= 1-point PGI-S improvement)	98	1.3 (1.07)	1 (-1, 4)	1.770 (<.001)	<.001	<.001	
  Stable (0-point PGI-S change) [reference]	86	0.5 (0.88)	0 (-1, 3)	0.667 (<.001)			
  Worsened (>= 1-point PGI-S worsening)	41	0.1 (0.86)	0 (-2, 3)	0.114 (0.472)	0.011		
	

Target Score / Anchor Groups	n	Mean change
(SD)	Median change
(min, max)	Within groups effect size (p-value)	Pair-wise comparisons p-value	One-way ANOVA p-value	
Near Vision Satisfaction	
  Improved (>= 2-point PGI-S improvement)	18	1.4 (1.14)	1 (0, 3)	1.620 (<.001)	0.005	<.001	
  Stable (<2-point PGI-S change) [reference]	207	0.5 (0.75)	0 (-1, 3)	0.676 (<.001)			
  Worsened (>= 2-point PGI-S worsening)	7	0.1 (1.35)	0 (-1, 2)	0.267 (0.788)	0.491		
	

Target Score / Anchor Groups	n	Mean change
(SD)	Median change
(min, max)	Within groups effect size (p-value)	Pair-wise comparisons p-value	One-way ANOVA p-value	
Near Vision Satisfaction	
  Improved (>= 2-point PGI-S improvement)	30	1.6 (0.96)	2 (-1, 3)	2.397 (<.001)	<.001	<.001	
  Stable (<2-point PGI-S change) [reference]	189	0.6 (0.87)	0 (-1, 3)	0.741 (<.001)			
  Worsened (>= 2-point PGI-S worsening)	8	-0.4 (1.19)	0 (-2, 1)	-0.409 (0.402)	0.061		
	

Target Score / Anchor Groups	n	Mean change
(SD)	Median change
(min, max)	Within groups effect size (p-value)	Pair-wise comparisons p-value	One-way ANOVA p-value	
Near Vision Satisfaction	
  Improved (>= 2-point PGI-S improvement)	27	2.0 (1.07)	2 (-1, 4)	2.498 (<.001)	<.001	<.001	
  Stable (<2-point PGI-S change) [reference]	192	0.6 (0.93)	0 (-1, 3)	0.821 (<.001)			
  Worsened (>= 2-point PGI-S worsening)	6	0.2 (1.72)	0 (-2, 3)	0.323 (0.822)	0.540		
	

Target Score / Anchor Groups	n	Mean change
(SD)	Median change
(min, max)	Within groups effect size (p-value)	Pair-wise comparisons p-value	One-way ANOVA p-value	
Near Vision Satisfaction	
  4-point PGI-S improvement	0	-	-	-	-	<.001	
  3-point PGI-S improvement	2	1.5 (2.12)	2 (0, 3)	1.061 (0.500)	0.584	-	
  2-point PGI-S improvement	16	1.4 (1.09)	1 (0, 3)	1.689 (<.001)	0.002	-	
  1-point PGI-S improvement	71	0.9 (0.79)	1 (0, 3)	1.050 (<.001)	<.001	-	
  0-point PGI-S change [reference]	102	0.4 (0.61)	0 (-1, 2)	0.498 (<.001)			
  1-point PGI-S worsening	34	0.2 (0.72)	0 (-1, 3)	0.273 (0.160)	0.203		
  2-point PGI-S worsening	6	0.3 (1.37)	0 (-1, 2)	0.645 (0.576)	0.973	-	
  3-point PGI-S worsening	1	-1.0 (-)	-1 (-1, -1)	-	-	-	
  4-point PGI-S worsening	0	-	-	-	-	-	
	

Target Score / Anchor Groups	n	Mean change
(SD)	Median change
(min, max)	Within groups effect size (p-value)	Pair-wise comparisons p-value	One-way ANOVA p-value	
Near Vision Satisfaction	
  4-point PGI-S improvement	0	-	-	-	-	<.001	
  3-point PGI-S improvement	1	3.0 (-)	3 (3, 3)	-	-	-	
  2-point PGI-S improvement	29	1.6 (0.95)	2 (-1, 3)	2.307 (<.001)	<.001	-	
  1-point PGI-S improvement	70	0.9 (0.85)	1 (-1, 3)	1.261 (<.001)	<.001	-	
  0-point PGI-S change [reference]	88	0.4 (0.84)	0 (-1, 3)	0.547 (<.001)			
  1-point PGI-S worsening	31	0.1 (0.67)	0 (-1, 2)	0.159 (0.292)	0.056		
  2-point PGI-S worsening	6	0.2 (0.75)	0 (-1, 1)	0.204 (0.611)	0.459	-	
  3-point PGI-S worsening	2	-2.0 (0.00)	-2 (-2, -2)	-2.828 (<.001)	<.001	-	
  4-point PGI-S worsening	0	-	-	-	-	-	
	

Target Score / Anchor Groups	n	Mean change
(SD)	Median change
(min, max)	Within groups effect size (p-value)	Pair-wise comparisons p-value	One-way ANOVA p-value	
Near Vision Satisfaction	
  4-point PGI-S improvement	0	-	-	-	-	<.001	
  3-point PGI-S improvement	4	2.0 (1.41)	2 (1, 4)	4.000 (0.066)	0.127	-	
  2-point PGI-S improvement	23	2.0 (1.04)	2 (-1, 3)	2.384 (<.001)	<.001	-	
  1-point PGI-S improvement	71	1.0 (0.94)	1 (-1, 3)	1.447 (<.001)	<.001	-	
  0-point PGI-S change [reference]	86	0.5 (0.88)	0 (-1, 3)	0.667 (<.001)			
  1-point PGI-S worsening	35	0.1 (0.66)	0 (-1, 2)	0.100 (0.447)	0.004		
  2-point PGI-S worsening	5	0.6 (1.52)	0 (-1, 3)	1.342 (0.426)	0.916	-	
  3-point PGI-S worsening	1	-2.0 (-)	-2 (-2, -2)	-	-	-	
  4-point PGI-S worsening	0	-	-	-	-	-	
	

Target Score / Anchor Groups	n	Mean change
(SD)	Median change
(min, max)	Within groups effect size (p-value)	Pair-wise comparisons p-value	One-way ANOVA p-value	
Near Vision Satisfaction	
  Improved (A little better/Much better)	82	1.1 (0.87)	1 (-1, 3)	1.470 (<.001)	<.001	<.001	
  Stable (No change) [reference]	139	0.3 (0.61)	0 (-1, 3)	0.323 (<.001)			
  Worsened (A little worse/Much worse)	11	0.5 (1.13)	0 (-1, 2)	0.843 (0.140)	0.424		
	

Target Score / Anchor Groups	n	Mean change
(SD)	Median change
(min, max)	Within groups effect size (p-value)	Pair-wise comparisons p-value	One-way ANOVA p-value	
Near Vision Satisfaction	
  Improved (A little better/Much better)	94	1.2 (0.94)	1 (-1, 3)	1.513 (<.001)	<.001	<.001	
  Stable (No change) [reference]	120	0.3 (0.76)	0 (-2, 3)	0.346 (<.001)			
  Worsened (A little worse/Much worse)	12	0.3 (1.15)	0 (-1, 3)	0.442 (0.339)	0.848		
	

Target Score / Anchor Groups	n	Mean change
(SD)	Median change
(min, max)	Within groups effect size (p-value)	Pair-wise comparisons p-value	One-way ANOVA p-value	
Near Vision Satisfaction	
  Improved (A little better/Much better)	99	1.4 (1.02)	1 (0, 4)	1.779 (<.001)	<.001	<.001	
  Stable (No change) [reference]	113	0.3 (0.74)	0 (-2, 3)	0.356 (<.001)			
  Worsened (A little worse/Much worse)	13	0.3 (1.11)	0 (-1, 3)	0.332 (0.337)	0.874		
	

Target Score / Anchor Groups	n	Mean change
(SD)	Median change
(min, max)	Within groups effect size (p-value)	Pair-wise comparisons p-value	One-way ANOVA p-value	
Near Vision Satisfaction	
  Improved (>= 0.14 logMAR decrease)	65	0.8 (0.85)	1 (0, 3)	1.127 (<.001)	0.002	0.001	
  Stable (<0.14 absolute logMAR change) [reference]	165	0.5 (0.81)	0 (-1, 3)	0.574 (<.001)			
  Worsened (>= 0.14 logMAR increase)	0	-	-	-	-		
	

Target Score / Anchor Groups	n	Mean change
(SD)	Median change
(min, max)	Within groups effect size (p-value)	Pair-wise comparisons p-value	One-way ANOVA p-value	
Near Vision Satisfaction	
  Improved (>= 0.14 logMAR decrease)	78	1.1 (1.00)	1 (-1, 3)	1.450 (<.001)	<.001	<.001	
  Stable (<0.14 absolute logMAR change) [reference]	143	0.5 (0.93)	0 (-2, 3)	0.597 (<.001)			
  Worsened (>= 0.14 logMAR increase)	3	0.0 (0.00)	0 (0, 0)	0.000 (.)	<.001		
	

Target Score / Anchor Groups	n	Mean change
(SD)	Median change
(min, max)	Within groups effect size (p-value)	Pair-wise comparisons p-value	One-way ANOVA p-value	
Near Vision Satisfaction	
  Improved (>= 0.14 logMAR decrease)	83	1.1 (1.13)	1 (-1, 4)	1.342 (<.001)	<.001	<.001	
  Stable (<0.14 absolute logMAR change) [reference]	138	0.6 (0.97)	0 (-2, 3)	0.762 (<.001)			
  Worsened (>= 0.14 logMAR increase)	1	2.0 (-)	2 (2, 2)	-	-		
	

Target Score / Anchor Groups	n	Mean change
(SD)	Median change
(min, max)	Within groups effect size (p-value)	Pair-wise comparisons p-value	One-way ANOVA p-value	
Near Vision Correction Independence	
  Improved (>= 1-point PGI-S improvement)	89	-0.7 (1.08)	-1 (-3, 4)	-0.711 (<.001)	<.001	<.001	
  Stable (0-point PGI-S change) [reference]	102	-0.3 (0.66)	0 (-2, 2)	-0.261 (<.001)			
  Worsened (>= 1-point PGI-S worsening)	41	0.1 (0.79)	0 (-2, 3)	0.162 (0.244)	0.005		
	

Target Score / Anchor Groups	n	Mean change
(SD)	Median change
(min, max)	Within groups effect size (p-value)	Pair-wise comparisons p-value	One-way ANOVA p-value	
Near Vision Correction Independence	
  Improved (>= 1-point PGI-S improvement)	100	-0.8 (1.09)	-1 (-4, 3)	-0.865 (<.001)	<.001	<.001	
  Stable (0-point PGI-S change) [reference]	88	-0.1 (0.63)	0 (-2, 2)	-0.142 (0.045)			
  Worsened (>= 1-point PGI-S worsening)	39	0.3 (0.79)	0 (-2, 3)	0.233 (0.048)	0.008		
	

Target Score / Anchor Groups	n	Mean change
(SD)	Median change
(min, max)	Within groups effect size (p-value)	Pair-wise comparisons p-value	One-way ANOVA p-value	
Near Vision Correction Independence	
  Improved (>= 1-point PGI-S improvement)	98	-0.8 (1.16)	-1 (-4, 4)	-0.766 (<.001)	<.001	<.001	
  Stable (0-point PGI-S change) [reference]	86	-0.2 (0.61)	0 (-2, 1)	-0.227 (0.003)			
  Worsened (>= 1-point PGI-S worsening)	41	0.2 (0.80)	0 (-1, 3)	0.233 (0.058)	0.003		
	

Target Score / Anchor Groups	n	Mean change
(SD)	Median change
(min, max)	Within groups effect size (p-value)	Pair-wise comparisons p-value	One-way ANOVA p-value	
Near Vision Correction Independence	
  Improved (>= 2-point PGI-S improvement)	18	-0.9 (1.30)	-1 (-3, 1)	-1.023 (0.007)	0.069	0.009	
  Stable (<2-point PGI-S change) [reference]	207	-0.3 (0.87)	0 (-3, 4)	-0.333 (<.001)			
  Worsened (>= 2-point PGI-S worsening)	7	0.1 (0.69)	0 (-1, 1)	0.189 (0.604)	0.118		
	

Target Score / Anchor Groups	n	Mean change
(SD)	Median change
(min, max)	Within groups effect size (p-value)	Pair-wise comparisons p-value	One-way ANOVA p-value	
Near Vision Correction Independence	
  Improved (>= 2-point PGI-S improvement)	30	-1.4 (1.10)	-2 (-4, 2)	-1.617 (<.001)	<.001	<.001	
  Stable (<2-point PGI-S change) [reference]	189	-0.2 (0.85)	0 (-3, 3)	-0.231 (<.001)			
  Worsened (>= 2-point PGI-S worsening)	8	-0.1 (0.99)	0 (-2, 1)	-0.071 (0.732)	0.792		
	

Target Score / Anchor Groups	n	Mean change
(SD)	Median change
(min, max)	Within groups effect size (p-value)	Pair-wise comparisons p-value	One-way ANOVA p-value	
Near Vision Correction Independence	
  Improved (>= 2-point PGI-S improvement)	27	-1.3 (1.18)	-1 (-4, 1)	-1.337 (<.001)	<.001	<.001	
  Stable (<2-point PGI-S change) [reference]	192	-0.3 (0.91)	0 (-3, 4)	-0.280 (<.001)			
  Worsened (>= 2-point PGI-S worsening)	6	0.3 (0.52)	0 (0, 1)	0.373 (0.175)	0.032		
	

Target Score / Anchor Groups	n	Mean change
(SD)	Median change
(min, max)	Within groups effect size (p-value)	Pair-wise comparisons p-value	One-way ANOVA p-value	
Near Vision Correction Independence	
  4-point PGI-S improvement	0	-	-	-	-	<.001	
  3-point PGI-S improvement	2	-2.0 (0.00)	-2 (-2, -2)	-2.828 (<.001)	<.001	-	
  2-point PGI-S improvement	16	-0.8 (1.33)	-1 (-3, 1)	-0.849 (0.027)	0.125	-	
  1-point PGI-S improvement	71	-0.7 (1.03)	-1 (-3, 4)	-0.640 (<.001)	0.004	-	
  0-point PGI-S change [reference]	102	-0.3 (0.66)	0 (-2, 2)	-0.261 (<.001)			
  1-point PGI-S worsening	34	0.1 (0.82)	0 (-2, 3)	0.157 (0.304)	0.011		
  2-point PGI-S worsening	6	0.0 (0.63)	0 (-1, 1)	0.000 (1.000)	0.361	-	
  3-point PGI-S worsening	1	1.0 (-)	1 (1, 1)	-	-	-	
  4-point PGI-S worsening	0	-	-	-	-	-	
	

Target Score / Anchor Groups	n	Mean change
(SD)	Median change
(min, max)	Within groups effect size (p-value)	Pair-wise comparisons p-value	One-way ANOVA p-value	
Near Vision Correction Independence	
  4-point PGI-S improvement	0	-	-	-	-	<.001	
  3-point PGI-S improvement	1	-4.0 (-)	-4 (-4, -4)	-	-	-	
  2-point PGI-S improvement	29	-1.3 (1.01)	-1 (-3, 2)	-1.512 (<.001)	<.001	-	
  1-point PGI-S improvement	70	-0.6 (0.99)	-1 (-3, 3)	-0.582 (<.001)	0.001	-	
  0-point PGI-S change [reference]	88	-0.1 (0.63)	0 (-2, 2)	-0.142 (0.045)			
  1-point PGI-S worsening	31	0.4 (0.71)	0 (-1, 3)	0.413 (0.009)	0.001		
  2-point PGI-S worsening	6	-0.3 (1.03)	0 (-2, 1)	-0.199 (0.465)	0.663	-	
  3-point PGI-S worsening	2	0.5 (0.71)	1 (0, 1)	0.236 (0.500)	0.421	-	
  4-point PGI-S worsening	0	-	-	-	-	-	
	

Target Score / Anchor Groups	n	Mean change
(SD)	Median change
(min, max)	Within groups effect size (p-value)	Pair-wise comparisons p-value	One-way ANOVA p-value	
Near Vision Correction Independence	
  4-point PGI-S improvement	0	-	-	-	-	<.001	
  3-point PGI-S improvement	4	-1.3 (2.22)	-1 (-4, 1)	-2.165 (0.342)	0.413	-	
  2-point PGI-S improvement	23	-1.3 (0.98)	-1 (-3, 1)	-1.290 (<.001)	<.001	-	
  1-point PGI-S improvement	71	-0.6 (1.10)	-1 (-3, 4)	-0.569 (<.001)	0.004	-	
  0-point PGI-S change [reference]	86	-0.2 (0.61)	0 (-2, 1)	-0.227 (0.003)			
  1-point PGI-S worsening	35	0.2 (0.84)	0 (-1, 3)	0.212 (0.118)	0.009		
  2-point PGI-S worsening	5	0.2 (0.45)	0 (0, 1)	0.200 (0.374)	0.119	-	
  3-point PGI-S worsening	1	1.0 (-)	1 (1, 1)	-	-	-	
  4-point PGI-S worsening	0	-	-	-	-	-	
	

Target Score / Anchor Groups	n	Mean change
(SD)	Median change
(min, max)	Within groups effect size (p-value)	Pair-wise comparisons p-value	One-way ANOVA p-value	
Near Vision Correction Independence	
  Improved (A little better/Much better)	82	-0.8 (0.99)	-1 (-3, 4)	-0.832 (<.001)	<.001	<.001	
  Stable (No change) [reference]	139	-0.1 (0.74)	0 (-3, 3)	-0.079 (0.211)			
  Worsened (A little worse/Much worse)	11	-0.5 (1.04)	0 (-2, 1)	-0.522 (0.111)	0.172		
	

Target Score / Anchor Groups	n	Mean change
(SD)	Median change
(min, max)	Within groups effect size (p-value)	Pair-wise comparisons p-value	One-way ANOVA p-value	
Near Vision Correction Independence	
  Improved (A little better/Much better)	94	-0.8 (1.04)	-1 (-3, 3)	-0.896 (<.001)	<.001	<.001	
  Stable (No change) [reference]	120	-0.0 (0.67)	0 (-2, 3)	-0.025 (0.682)			
  Worsened (A little worse/Much worse)	12	-0.2 (1.53)	0 (-4, 1)	-0.150 (0.713)	0.756		
	

Target Score / Anchor Groups	n	Mean change
(SD)	Median change
(min, max)	Within groups effect size (p-value)	Pair-wise comparisons p-value	One-way ANOVA p-value	
Near Vision Correction Independence	
  Improved (A little better/Much better)	99	-0.9 (0.99)	-1 (-4, 1)	-0.930 (<.001)	<.001	<.001	
  Stable (No change) [reference]	113	0.0 (0.74)	0 (-1, 4)	0.036 (0.614)			
  Worsened (A little worse/Much worse)	13	0.2 (0.99)	0 (-2, 2)	0.171 (0.584)	0.682		
	

Target Score / Anchor Groups	n	Mean change
(SD)	Median change
(min, max)	Within groups effect size (p-value)	Pair-wise comparisons p-value	One-way ANOVA p-value	
Near Vision Correction Independence	
  Improved (>= 0.14 logMAR decrease)	65	-0.6 (1.07)	-1 (-3, 4)	-0.658 (<.001)	0.012	0.005	
  Stable (<0.14 absolute logMAR change) [reference]	165	-0.3 (0.84)	0 (-3, 3)	-0.263 (<.001)			
  Worsened (>= 0.14 logMAR increase)	0	-	-	-	-		
	

Target Score / Anchor Groups	n	Mean change
(SD)	Median change
(min, max)	Within groups effect size (p-value)	Pair-wise comparisons p-value	One-way ANOVA p-value	
Near Vision Correction Independence	
  Improved (>= 0.14 logMAR decrease)	78	-0.7 (1.13)	-1 (-4, 3)	-0.666 (<.001)	0.001	<.001	
  Stable (<0.14 absolute logMAR change) [reference]	143	-0.2 (0.86)	0 (-3, 3)	-0.235 (0.002)			
  Worsened (>= 0.14 logMAR increase)	3	0.3 (0.58)	0 (0, 1)	0.289 (0.423)	0.233		
	

Target Score / Anchor Groups	n	Mean change
(SD)	Median change
(min, max)	Within groups effect size (p-value)	Pair-wise comparisons p-value	One-way ANOVA p-value	
Near Vision Correction Independence	
  Improved (>= 0.14 logMAR decrease)	83	-0.6 (1.10)	0 (-4, 4)	-0.613 (<.001)	0.008	0.020	
  Stable (<0.14 absolute logMAR change) [reference]	138	-0.3 (0.92)	0 (-3, 3)	-0.269 (0.002)			
  Worsened (>= 0.14 logMAR increase)	1	0.0 (-)	0 (0, 0)	-	-		
	
