# Supplementary material for: Psychometric evaluation of the near activity visual questionnaire presbyopia (NAVQ-P) and additional patient-reported outcome items
Source: J Patient Rep Outcomes. 2024 Apr 9;8:41. doi: 10.1186/s41687-024-00717-9 (PMC11004101; doi:10.1186/s41687-024-00717-9)
Supplement: Supplementary file 5 — Supplementary Material 5 [file 41687_2024_717_MOESM5_ESM.rtf]

	Change from Baseline
to Month 1	Change from Baseline
to Month 2	Change from Baseline
to Month 3	
Target Score /
Anchor Groups	n (%)	Mean Change (SD)	MID estimate	n (%)	Mean Change (SD)	MID estimate	n (%)	Mean Change (SD)	MID estimate	
NAVQ-P Total	
  Improved (>= 1-point PGI-S improvement)	86 (38.7%)	-8.0 (7.95)	5.4	99 (45.0%)	-9.3 (8.81)	6.9	93 (43.3%)	-12.1 (9.39)	8.5	
  Stable (0-point PGI-S change) [reference]	98 (44.1%)	-2.5 (6.14)	-	85 (38.6%)	-2.5 (6.21)	-	86 (40.0%)	-3.6 (6.45)	-	
  Worsened (>= 1-point PGI-S worsening)	38 (17.1%)	2.0 (5.04)	-	36 (16.4%)	1.5 (6.95)	-	36 (16.7%)	1.5 (6.15)	-	
	


	Change from Baseline
to Month 1	Change from Baseline
to Month 2	Change from Baseline
to Month 3	
Target Score /
Anchor Groups	n (%)	Mean Change (SD)	MID estimate	n (%)	Mean Change (SD)	MID estimate	n (%)	Mean Change (SD)	MID estimate	
NAVQ-P Total	
  Improved (>= 2-point PGI-S improvement)	17 (7.7%)	-8.3 (9.37)	4.5	29 (13.2%)	-13.8 (9.31)	9.9	24 (11.2%)	-19.8 (9.03)	14.8	
  Stable (<2-point PGI-S change) [reference]	199 (89.6%)	-3.8 (7.29)	-	185 (84.1%)	-3.9 (7.59)	-	185 (86.0%)	-5.1 (7.76)	-	
  Worsened (>= 2-point PGI-S worsening)	6 (2.7%)	5.2 (5.38)	-	6 (2.7%)	6.0 (7.16)	-	6 (2.8%)	6.2 (7.88)	-	
	

	Change from Baseline
to Month 1	Change from Baseline
to Month 2	Change from Baseline
to Month 3	
Target Score /
Anchor Groups	n (%)	Mean Change (SD)	MID estimate	n (%)	Mean Change (SD)	MID estimate	n (%)	Mean Change (SD)	MID estimate	
NAVQ-P Total	
  Improved (A little better/Much better)	78 (35.1%)	-9.0 (7.20)	8.2	90 (41.1%)	-9.2 (8.22)	7.4	94 (43.7%)	-12.0 (8.97)	10.2	
  Stable (No change) [reference]	133 (59.9%)	-0.8 (5.75)	-	118 (53.9%)	-1.7 (6.92)	-	109 (50.7%)	-1.8 (6.24)	-	
  Worsened (A little worse/Much worse)	11 (5.0%)	-4.5 (10.88)	-	11 (5.0%)	-3.5 (13.84)	-	12 (5.6%)	-4.3 (12.92)	-	
	

	Change from Baseline
to Month 1	Change from Baseline
to Month 2	Change from Baseline
to Month 3	
Target Score /
Anchor Groups	n (%)	Mean Change (SD)	MID estimate	n (%)	Mean Change (SD)	MID estimate	n (%)	Mean Change (SD)	MID estimate	
NAVQ-P Total	
  Improved (>= 0.14 logMAR decrease)	61 (27.7%)	-6.9 (8.17)	4.1	75 (34.6%)	-8.9 (8.43)	5.9	79 (37.3%)	-9.4 (10.28)	4.8	
  Stable (<0.14 absolute logMAR change) [reference]	159 (72.3%)	-2.7 (7.11)	-	139 (64.1%)	-3.0 (8.06)	-	132 (62.3%)	-4.6 (8.45)	-	
  Worsened (>= 0.14 logMAR increase)	0	-	-	3 (1.4%)	3.7 (6.35)	-	1 (0.5%)	-7.0 (.)	-	
	

	Change from Baseline
to Month 1	Change from Baseline
to Month 2	Change from Baseline
to Month 3	
Target Score /
Anchor Groups	n (%)	Mean Change (SD)	MID estimate	n (%)	Mean Change (SD)	MID estimate	n (%)	Mean Change (SD)	MID estimate	
Near Vision Satisfaction	
  Improved (>= 1-point PGI-S improvement)	89 (38.4%)	1.0 (0.89)	-0.7	100 (44.1%)	1.2 (0.94)	-0.7	98 (43.6%)	1.3 (1.07)	-0.8	
  Stable (0-point PGI-S change) [reference]	102 (44.0%)	0.4 (0.61)	-	88 (38.8%)	0.4 (0.84)	-	86 (38.2%)	0.5 (0.88)	-	
  Worsened (>= 1-point PGI-S worsening)	41 (17.7%)	0.2 (0.83)	-	39 (17.2%)	0.0 (0.81)	-	41 (18.2%)	0.1 (0.86)	-	
	

	Change from Baseline
to Month 1	Change from Baseline
to Month 2	Change from Baseline
to Month 3	
Target Score /
Anchor Groups	n (%)	Mean Change (SD)	MID estimate	n (%)	Mean Change (SD)	MID estimate	n (%)	Mean Change (SD)	MID estimate	
Near Vision Satisfaction	
  Improved (>= 2-point PGI-S improvement)	18 (7.8%)	1.4 (1.14)	-0.9	30 (13.2%)	1.6 (0.96)	-1.1	27 (12.0%)	2.0 (1.07)	-1.4	
  Stable (<2-point PGI-S change) [reference]	207 (89.2%)	0.5 (0.75)	-	189 (83.3%)	0.6 (0.87)	-	192 (85.3%)	0.6 (0.93)	-	
  Worsened (>= 2-point PGI-S worsening)	7 (3.0%)	0.1 (1.35)	-	8 (3.5%)	-0.4 (1.19)	-	6 (2.7%)	0.2 (1.72)	-	
	

	Change from Baseline
to Month 1	Change from Baseline
to Month 2	Change from Baseline
to Month 3	
Target Score /
Anchor Groups	n (%)	Mean Change (SD)	MID estimate	n (%)	Mean Change (SD)	MID estimate	n (%)	Mean Change (SD)	MID estimate	
Near Vision Satisfaction	
  Improved (A little better/Much better)	82 (35.3%)	1.1 (0.87)	-0.9	94 (41.6%)	1.2 (0.94)	-1.0	99 (44.0%)	1.4 (1.02)	-1.2	
  Stable (No change) [reference]	139 (59.9%)	0.3 (0.61)	-	120 (53.1%)	0.3 (0.76)	-	113 (50.2%)	0.3 (0.74)	-	
  Worsened (A little worse/Much worse)	11 (4.7%)	0.5 (1.13)	-	12 (5.3%)	0.3 (1.15)	-	13 (5.8%)	0.3 (1.11)	-	
	

	Change from Baseline
to Month 1	Change from Baseline
to Month 2	Change from Baseline
to Month 3	
Target Score /
Anchor Groups	n (%)	Mean Change (SD)	MID estimate	n (%)	Mean Change (SD)	MID estimate	n (%)	Mean Change (SD)	MID estimate	
Near Vision Satisfaction	
  Improved (>= 0.14 logMAR decrease)	65 (28.3%)	0.8 (0.85)	-0.4	78 (34.8%)	1.1 (1.00)	-0.6	83 (37.4%)	1.1 (1.13)	-0.6	
  Stable (<0.14 absolute logMAR change) [reference]	165 (71.7%)	0.5 (0.81)	-	143 (63.8%)	0.5 (0.93)	-	138 (62.2%)	0.6 (0.97)	-	
  Worsened (>= 0.14 logMAR increase)	0	-	-	3 (1.3%)	0.0 (0.00)	-	1 (0.5%)	2.0 (.)	-	
	

	Change from Baseline
to Month 1	Change from Baseline
to Month 2	Change from Baseline
to Month 3	
Target Score /
Anchor Groups	n (%)	Mean Change (SD)	MID estimate	n (%)	Mean Change (SD)	MID estimate	n (%)	Mean Change (SD)	MID estimate	
Near Vision Correction Independence	
  Improved (>= 1-point PGI-S improvement)	89 (38.4%)	-0.7 (1.08)	0.5	100 (44.1%)	-0.8 (1.09)	0.7	98 (43.6%)	-0.8 (1.16)	0.6	
  Stable (0-point PGI-S change) [reference]	102 (44.0%)	-0.3 (0.66)	-	88 (38.8%)	-0.1 (0.63)	-	86 (38.2%)	-0.2 (0.61)	-	
  Worsened (>= 1-point PGI-S worsening)	41 (17.7%)	0.1 (0.79)	-	39 (17.2%)	0.3 (0.79)	-	41 (18.2%)	0.2 (0.80)	-	
	

	Change from Baseline
to Month 1	Change from Baseline
to Month 2	Change from Baseline
to Month 3	
Target Score /
Anchor Groups	n (%)	Mean Change (SD)	MID estimate	n (%)	Mean Change (SD)	MID estimate	n (%)	Mean Change (SD)	MID estimate	
Near Vision Correction Independence	
  Improved (>= 2-point PGI-S improvement)	18 (7.8%)	-0.9 (1.30)	0.6	30 (13.2%)	-1.4 (1.10)	1.2	27 (12.0%)	-1.3 (1.18)	1.1	
  Stable (<2-point PGI-S change) [reference]	207 (89.2%)	-0.3 (0.87)	-	189 (83.3%)	-0.2 (0.85)	-	192 (85.3%)	-0.3 (0.91)	-	
  Worsened (>= 2-point PGI-S worsening)	7 (3.0%)	0.1 (0.69)	-	8 (3.5%)	-0.1 (0.99)	-	6 (2.7%)	0.3 (0.52)	-	
	

	Change from Baseline
to Month 1	Change from Baseline
to Month 2	Change from Baseline
to Month 3	
Target Score /
Anchor Groups	n (%)	Mean Change (SD)	MID estimate	n (%)	Mean Change (SD)	MID estimate	n (%)	Mean Change (SD)	MID estimate	
Near Vision Correction Independence	
  Improved (A little better/Much better)	82 (35.3%)	-0.8 (0.99)	0.8	94 (41.6%)	-0.8 (1.04)	0.8	99 (44.0%)	-0.9 (0.99)	1.0	
  Stable (No change) [reference]	139 (59.9%)	-0.1 (0.74)	-	120 (53.1%)	-0.0 (0.67)	-	113 (50.2%)	0.0 (0.74)	-	
  Worsened (A little worse/Much worse)	11 (4.7%)	-0.5 (1.04)	-	12 (5.3%)	-0.2 (1.53)	-	13 (5.8%)	0.2 (0.99)	-	
	

	Change from Baseline
to Month 1	Change from Baseline
to Month 2	Change from Baseline
to Month 3	
Target Score /
Anchor Groups	n (%)	Mean Change (SD)	MID estimate	n (%)	Mean Change (SD)	MID estimate	n (%)	Mean Change (SD)	MID estimate	
Near Vision Correction Independence	
  Improved (>= 0.14 logMAR decrease)	65 (28.3%)	-0.6 (1.07)	0.4	78 (34.8%)	-0.7 (1.13)	0.5	83 (37.4%)	-0.6 (1.10)	0.4	
  Stable (<0.14 absolute logMAR change) [reference]	165 (71.7%)	-0.3 (0.84)	-	143 (63.8%)	-0.2 (0.86)	-	138 (62.2%)	-0.3 (0.92)	-	
  Worsened (>= 0.14 logMAR increase)	0	-	-	3 (1.3%)	0.3 (0.58)	-	1 (0.5%)	0.0 (.)	-	
	
