# Supplementary material for: Psychometric evaluation of the near activity visual questionnaire presbyopia (NAVQ-P) and additional patient-reported outcome items
Source: J Patient Rep Outcomes. 2024 Apr 9;8:41. doi: 10.1186/s41687-024-00717-9 (PMC11004101; doi:10.1186/s41687-024-00717-9)
Supplement: Supplementary file 6 — Supplementary Material 6 [file 41687_2024_717_MOESM6_ESM.docx]

| Table 12. Distribution-based responder definitions for NAVQ-P total score, NVCI and NVS using Baseline scores | | | |
| --- | --- | --- | --- |
| **Target Score** | **Randomized population (N=235)** | | |
|  | **n** | **0.5 SD** | **SEM** |
| NAVQ-P total score | 229 | 4.842 | 1.936 |
| NVCI | 234 | 0.500 | - |
| NVS | 234 | 0.388 | - |
| SD: Standard Deviation; SEM: Standard Error of Measurement. 0.5 SD corresponds to 0.5 of the standard deviation at Baseline. The SEM is calculated as the standard deviation at Baseline multiplied by the square root of one minus the reliability (Cronbach's alpha) of the total score at Baseline. SEM was not calculated for the NVCI and NVS. | | | |
