# Supplementary material for: Psychometric evaluation of the near activity visual questionnaire presbyopia (NAVQ-P) and additional patient-reported outcome items
Source: J Patient Rep Outcomes. 2024 Apr 9;8:41. doi: 10.1186/s41687-024-00717-9 (PMC11004101; doi:10.1186/s41687-024-00717-9)
Supplement: Supplementary file 7 — Supplementary Material 7 [file 41687_2024_717_MOESM7_ESM.docx]

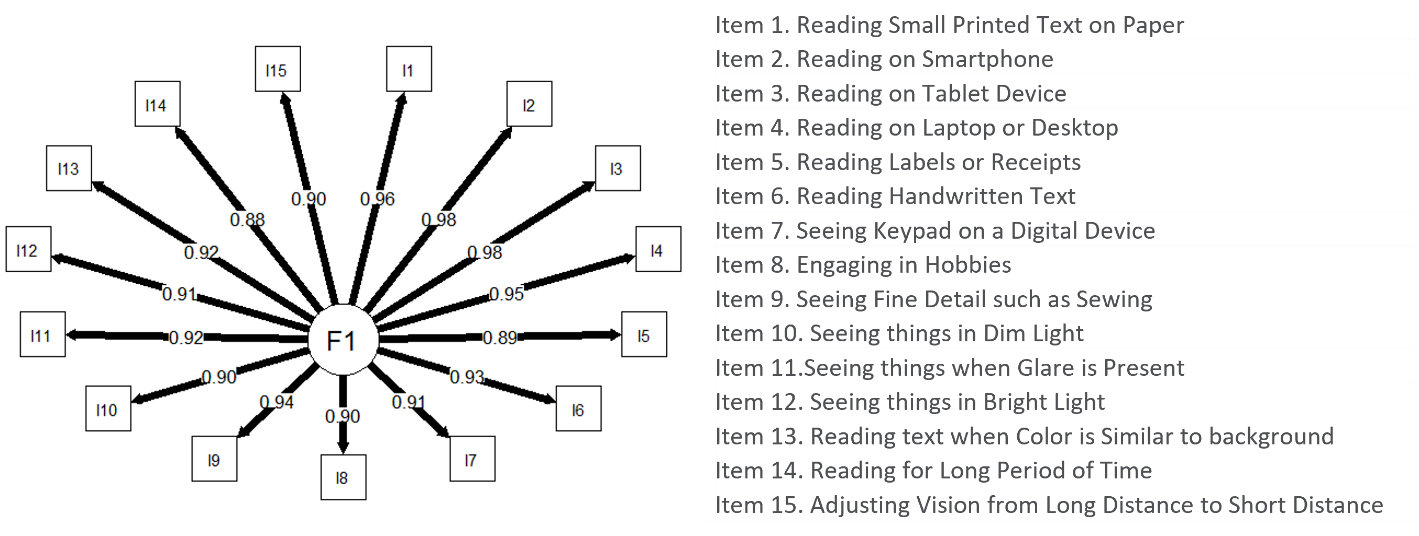
**
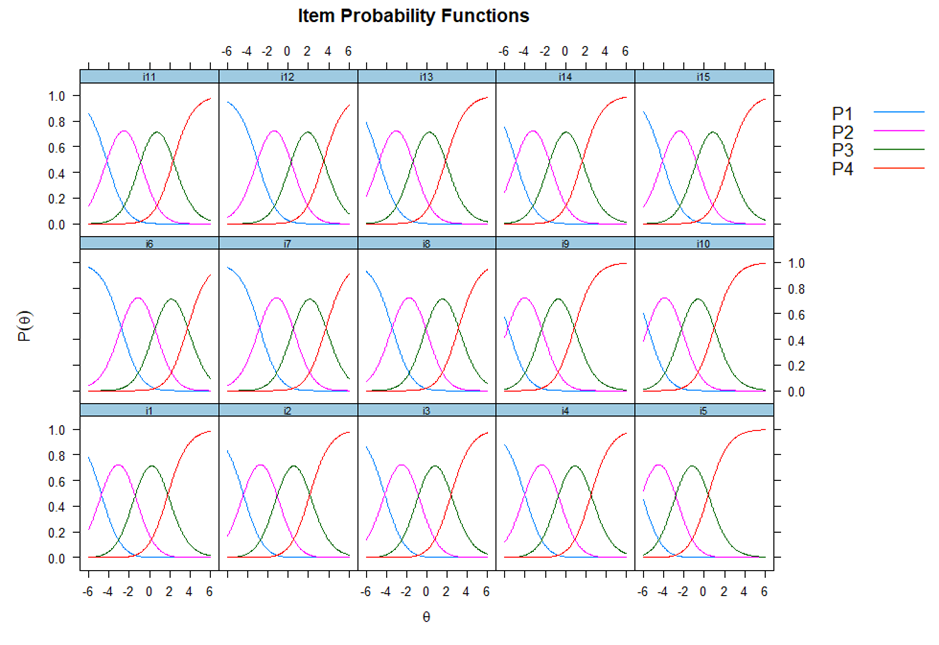
**

Figure 1. Item characteristic curves for the item responses for all NAVQ-P items in the cross-sectional analysis population at Month 2

Note, Theta (θ) represents the participant location on the latent trait (near vision functioning severity) with higher scores indicating more severe near vision functioning for individuals with presbyopia. The coloured curves represent the probability of participant endorsement at each response category/option as a function of Theta, with P1 the least severe response option (“No difficulty”) and P4 the most severe (“Extreme difficulty”). Each plot represents an item for the NAVQ-P (item number is indicated in the blue ribbon above each plot). Note that the threshold structure is fixed across all items due to the constraints of the RSM but items can move along the logit (θ) scale as items still have different difficulty parameters.
