# Supplementary material for: Psychometric evaluation of the near activity visual questionnaire presbyopia (NAVQ-P) and additional patient-reported outcome items
Source: J Patient Rep Outcomes. 2024 Apr 9;8:41. doi: 10.1186/s41687-024-00717-9 (PMC11004101; doi:10.1186/s41687-024-00717-9)
Supplement: Supplementary file 8 — Supplementary Material 8 [file 41687_2024_717_MOESM8_ESM.rtf]

Local dependency evaluation (cross-sectional analysis population, Month 2)
Item	i1	i2	i3	i4	i5	i6	i7	i8	i9	i10	i11	i12	i13	i14	i15	
i1	1	-	-	-	-	-	-	-	-	-	-	-	-	-	-	
i2	0.28	1	-	-	-	-	-	-	-	-	-	-	-	-	-	
i3	-	0.51	1	-	-	-	-	-	-	-	-	-	-	-	-	
i4	-	0.31	0.56	1	-	-	-	-	-	-	-	-	-	-	-	
i5	-	-	-	-	1	-	-	-	-	-	-	-	-	-	-	
i6	-	-	-	-	-	1	-	-	-	-	-	-	-	-	-	
i7	-	-	-	-	-	-	1	-	-	-	-	-	-	-	-	
i8	-	-	-	-	-	-	-	1	-	-	-	-	-	-	-	
i9	-	-	-	-	-	-	-	-	1	-	-	-	-	-	-	
i10	-	-	-	-	-	-	-	-	-	1	-	-	-	-	-	
i11	-	-	-	-	-	-	-	-	-	-	1	-	-	-	-	
i12	-	-	-	-	-	-	-	-	-	-	-	1	-	-	-	
i13	-	-	-	-	-	-	-	-	-	-	-	-	1	-	-	
i14	-	-	-	-	-	-	-	-	-	-	-	-	-	1	-	
i15	-	-	-	-	-	-	-	-	-	-	-	-	-	0.24	1	
Off-diagonal elements shown in the table are Yen Q3 statistics which exceed thresholds (average residual correlation; -0.066 + 0.30) for local dependency.
i1 to i15 represent item 1 to item 15.
