# Supplementary material for: Psychometric evaluation of the near activity visual questionnaire presbyopia (NAVQ-P) and additional patient-reported outcome items
Source: J Patient Rep Outcomes. 2024 Apr 9;8:41. doi: 10.1186/s41687-024-00717-9 (PMC11004101; doi:10.1186/s41687-024-00717-9)
Supplement: Supplementary file 11 — Supplementary Material 11 [file 41687_2024_717_MOESM11_ESM.docx]

| **Table 1. Inclusion and exclusion criteria for trial population** | |
| --- | --- |
| Inclusion/exclusion criteria | |
| **Inclusion criteria** | |
| 1 | Written informed consent must be obtained before any assessment is performed. |
| 2 | 2. Phakic male or female participants aged 45 to 55 years, inclusive, at the Screening visit. |
| 3 | Monocular and binocular distance-corrected near visual acuity at 40 cm distance worse than 0.3 logMAR at the Screening and Baseline visits. |
| 4 | Binocular distance-corrected near visual acuity at baseline must not be different by more than 0.1 logMAR from the corresponding assessment at the Screening visit. |
| 5 | Need a minimum near addition prescription of +1.00 D or more to achieve binocular DCNVA of at least 0.0 logMAR at 40 cm distance, as assessed by the Investigator, at the Screening visit. |
| **Exclusion criteria** | |
| 1 | Best-corrected distance visual acuity worse than 0.0 logMAR at 4 m distance in either eye at the Screening visit. |
| 2 | Spherical equivalent greater than +4.0 D or less than -4.0 D, based on manifest refraction, in either eye at the Screening visit. |
| 3 | Astigmatism of greater than 1.25 D, based on manifest refraction, in either eye at the Screening visit. |
| 4 | Difference of greater than 0.50D between manifest refraction spherical equivalent and the cycloplegic refraction spherical equivalent. |
| 5 | Difference in manifest refraction spherical equivalent of greater than 0.75 D between eyes at the Screening visit. |
| 6 | Unequal pupil diameters with a difference of greater than 1 mm between eyes. |
| 7 | Non-circular pupil assessed by the Investigator to be related to a pathologic cause. |
| 8 | Contraindication to pupil dilation in either eye or a history of untreated narrow angles or currently occludable angles in either eye. |
| 9 | Insufficient pupillary dilation that precludes observation of the fundus or lens in either eye, in the opinion of the investigator. |
| 10 | Contact lens wear within 1 week for soft lenses and 2 weeks for hard or toric lenses, prior to Screening visit and for the duration of the study. |
| 11 | Prior history or current diagnosis of accommodative spasm, accommodative insufficiencies or other accommodative issues, except age-related accommodative issues related to presbyopia. |
| 12 | Any clinically significant congenital malformation or acquired changes to the lens or iris in either eye that might have an impact on visual acuity (e.g., clinically significant cataractous lens changes) or clinically significant phacodonesis. |
| 13 | Secondary cause of presbyopia in either eye (e.g., damage to lens, zonules or ciliary muscle, multiple sclerosis, and myasthenia gravis). |
| 14 | Any active ocular infection (i.e., bacterial, viral, parasitic or fungal), or inflammation, or a history of herpetic ocular infection in either eye at the Screening or Baseline visit. |
| 15 | History of idiopathic or auto-immune uveitis in either eye. |
| 16 | Ocular surface disease with a IDEEL blurry vision score greater than "slightly" AND corneal staining in the central zone greater than grade 1 at Screening visit. |
| 17 | History or current diagnosis of treated or untreated glaucoma of any type. |
| 18 | History of penetrating ocular trauma, significant blunt ocular trauma or uveitis in either eye. |
| 19 | Prior intraocular/extraocular surgery or laser surgery of any kind in either eye, including cataract surgery in either eye during the study period. |
| 20 | History of hypersensitivity to any of the study treatments (including placebo) or its inactive ingredients or to active ingredients of similar chemical classes. |
| 21 | Change in dose of any medication known to affect accommodation, pupil size or intraocular pressure during the study, as listed in Table 6-3. Participants who have been on This document (090095af8e0c252f in docbase CREDI_EH) has been digitally signed with external signatures using Entrust PKI. Signatures manifested as of 11/9/2020 1:33:53 PM, signing status at this time: Completed (1 of 1 signatures) Approved for report publication by Carten James in East Hanover at Mon, 09 Nov 2020 08:33:36 AM EST Novartis Confidential Page 22 of 74 Clinical Trial Protocol (Version 00) Protocol No. CUNR844A2202 stable dose of such medications for at least three months before the Screening visit and who are not expected to change the dose/ regimen or discontinue the medication are eligible for the study. |
| 22 | Use of other investigational drugs within 5 half-lives or within 30 days of the Screening visit, until the expected pharmacodynamic effect has returned to baseline, whichever is longer. |
| 23 | Prior therapy for presbyopia other than physical optical correction (e.g., supplements, medications, training exercises, ciliary body electrostimulation, corneal implants surgery). |
| 24 | History of clinically significant cardiac abnormalities or cerebrovascular conditions. |
| 25 | Suboptimally controlled diabetes mellitus (i.e., HbA1c 7% or more at the Screening visit) or history of insulin autoimmune disease. |
| 26 | History of or current use of arlipoic acid or dihydrolipoic acid oral supplements or eye drops. |
| 27 | Prior participation in a clinical study evaluating UNR844 or EV06. |
| 28 | History of malignancy of any organ system (other than localized squamous cell or basal cell carcinoma of the skin or in situ cervical cancer) within six months of the Screening visit. |
| 29 | Any ocular or systemic condition that, in the opinion of the Investigator, would jeopardize subject safety, has an impact on visual acuity, affects study assessments or validity of study results. |
| 30 | Pregnant or nursing (lactating) women |
| 31 | Women of child-bearing potential, defined as all women physiologically capable of becoming pregnant, unless they are using basic methods of contraception during dosing of investigational drug. Basic contraception methods include: · Total abstinence (when this is in line with the preferred and usual lifestyle of the participant. Periodic abstinence (e.g., calendar, ovulation, symptothermal, postovulation methods) and withdrawal are not acceptable methods of contraception · Female sterilization (have had surgical bilateral oophorectomy with or without hysterectomy), total hysterectomy or bilateral tubal ligation at least six weeks before taking investigational drug. In case of oophorectomy alone, only when the reproductive status of the woman has been confirmed by follow up hormone level assessment · Male sterilization (at least 6 months prior to screening). For female participants on the study, the vasectomized male partner should be the sole partner for that subject. Barrier methods of contraception: Condom or Occlusive cap (diaphragm or cervical/vault caps). For UK: with spermicidal foam/gel/film/cream/ vaginal suppository · Use of oral, (estrogen and progesterone), injected or implanted hormonal methods of contraception or other forms of hormonal contraception that have comparable efficacy (failure rate <1%), for example hormone vaginal ring or transdermal hormone contraception or placement of an intrauterine device (IUD) or intrauterine system (IUS). In case of use of oral contraception women should have been stable on the same pill for a minimum of 3 months before taking investigational drug. If local regulations deviate from the contraception methods listed above to prevent pregnancy, local regulations apply and will be described in the ICF. Women are considered post-menopausal and not of child bearing potential if they have had 12 months of natural (spontaneous) amenorrhea with an appropriate clinical profile (e.g. age appropriate, history of vasomotor symptoms) or have had surgical bilateral oophorectomy (with or without hysterectomy), total hysterectomy or bilateral tubal ligation at least six weeks ago. In the case of oophorectomy alone, only when the reproductive status of the woman has been confirmed by follow up hormone level assessment is she considered not of child bearing potential. |
| 32 | Participants in a dependent or unequal relationship with the Sponsor or study site staff (e.g., employees of the Sponsor, employees or students under the direct supervision of the study site staff, immediate relatives of the study site staff). |
