# Supplementary figures and images for: Psychometric evaluation of the near activity visual questionnaire presbyopia (NAVQ-P) and additional patient-reported outcome items
Source: J Patient Rep Outcomes. 2024 Apr 9;8:41. doi: 10.1186/s41687-024-00717-9 (PMC11004101; doi:10.1186/s41687-024-00717-9)

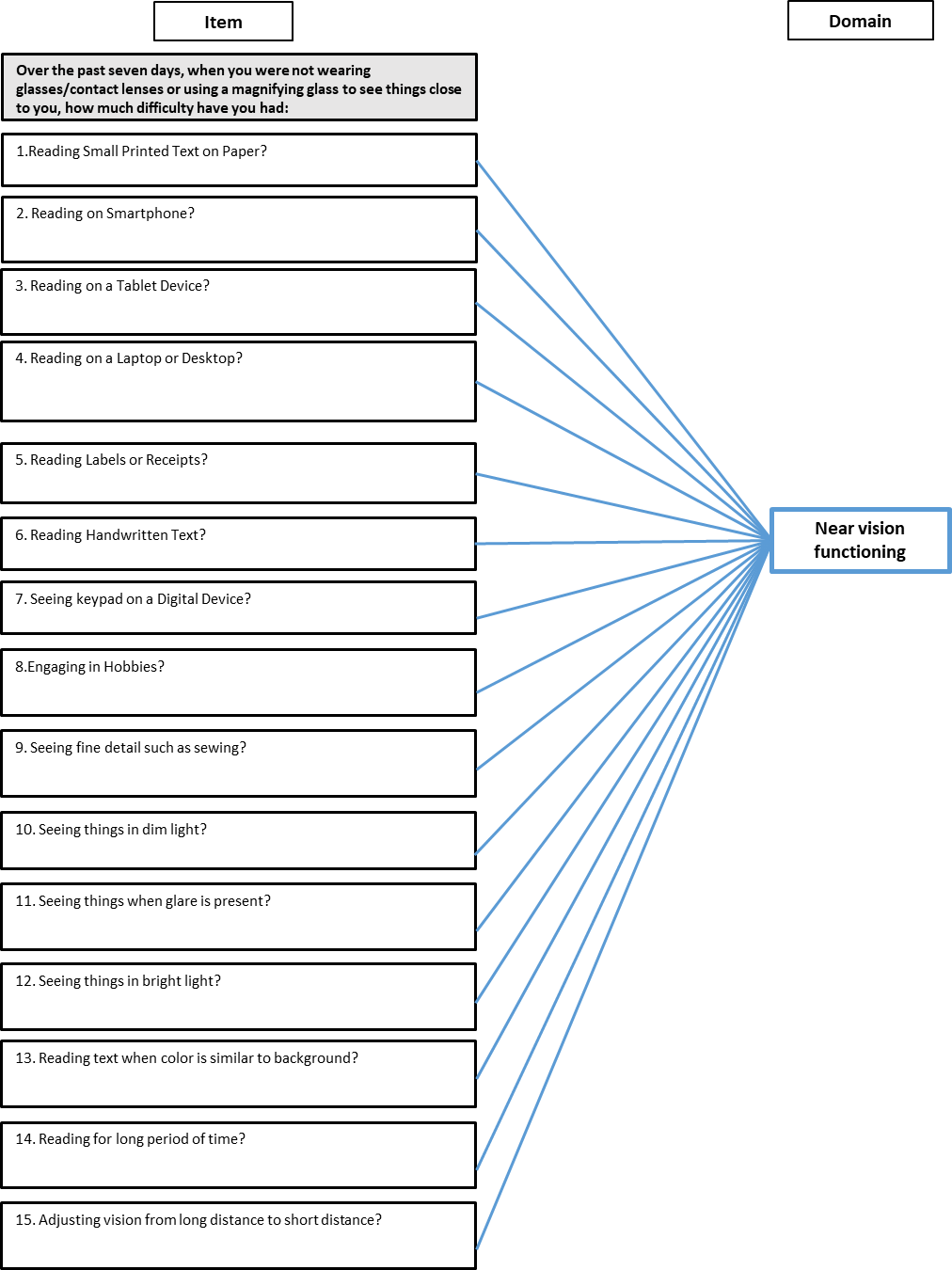


Figure 1. Proposed conceptual framework of the 15-item NAVQ-P

Note, only core item wording shown.

Supplement: Supplementary file 12 — Supplementary Material 12 [file 41687_2024_717_MOESM12_ESM.docx]

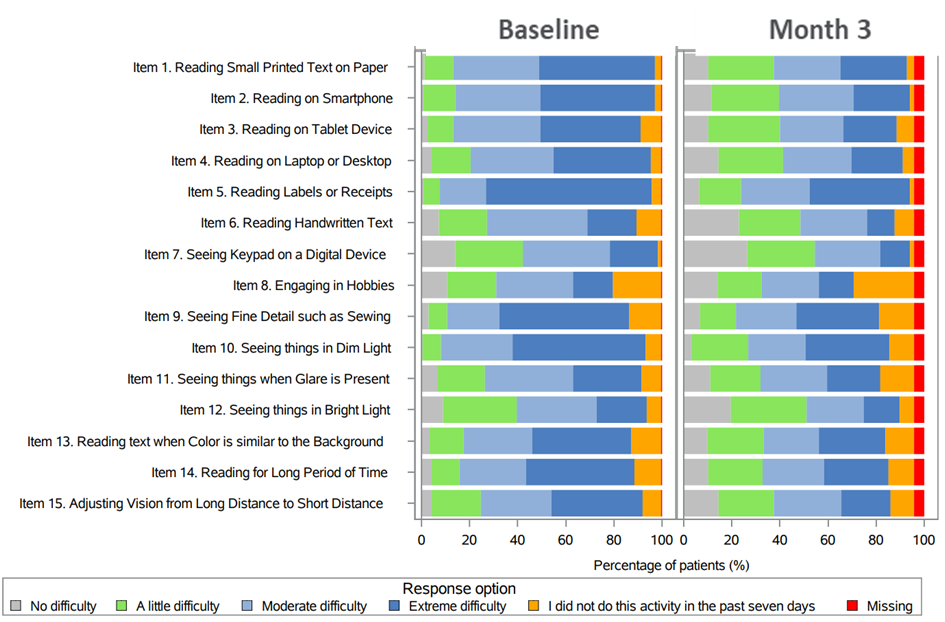


Figure 1. NAVQ-P item response distributions at Baseline and Month 3 (randomized population)

Supplement: Supplementary file 13 — Supplementary Material 13 [file 41687_2024_717_MOESM13_ESM.docx]
