# Supplementary material for: Psychometric evaluation of the near activity visual questionnaire presbyopia (NAVQ-P) and additional patient-reported outcome items
Source: J Patient Rep Outcomes. 2024 Apr 9;8:41. doi: 10.1186/s41687-024-00717-9 (PMC11004101; doi:10.1186/s41687-024-00717-9)
Supplement: Supplementary file 14 — Supplementary Material 14 [file 41687_2024_717_MOESM14_ESM.docx]

|  | **Randomized Population (N=235)** | | | | |
| --- | --- | --- | --- | --- | --- |
|  | **Baseline** | **Week 2** | **Month 1** | **Month 2** | **Month 3** |
| Near Vision Satisfaction (NVS) | | | | | |
| Very dissatisfied | 128 (54.5%) | 69 (29.4%) | 58 (24.7%) | 59 (25.1%) | 58 (24.7%) |
| Dissatisfied | 77 (32.8%) | 96 (40.9%) | 104 (44.3%) | 83 (35.3%) | 74 (31.5%) |
| Neither satisfied nor dissatisfied | 24 (10.2%) | 42 (17.9%) | 42 (17.9%) | 49 (20.9%) | 51 (21.7%) |
| Satisfied | 4 (1.7%) | 17 (7.2%) | 27 (11.5%) | 34 (14.5%) | 33 (14.0%) |
| Very satisfied | 1 (0.4%) | 2 (0.9%) | 1 (0.4%) | 2 (0.9%) | 9 (3.8%) |
| Missing/no response | 1 (0.4%) | 9 (3.8%) | 3 (1.3%) | 8 (3.4%) | 10 (4.3%) |
| Near Vision Correction Independence (NVCI) | | | | | |
| Never | 6 (2.6%) | 9 (3.8%) | 13 (5.5%) | 13 (5.5%) | 12 (5.1%) |
| Rarely | 9 (3.8%) | 11 (4.7%) | 21 (8.9%) | 23 (9.8%) | 28 (11.9%) |
| Sometimes | 41 (17.4%) | 52 (22.1%) | 51 (21.7%) | 49 (20.9%) | 46 (19.6%) |
| Often | 73 (31.1%) | 66 (28.1%) | 75 (31.9%) | 66 (28.1%) | 61 (26.0%) |
| Always | 105 (44.7%) | 88 (37.4%) | 72 (30.6%) | 76 (32.3%) | 78 (33.2%) |
| Missing/no response | 1 (0.4%) | 9 (3.8%) | 3 (1.3%) | 8 (3.4%) | 10 (4.3%) |
| Near Vision Correction Preference (NVCP)* | | | | | |
| The study treatment (eye drops) | 71 (30.2%) | 79 (33.6%) | 89 (37.9%) | 98 (41.7%) | 88 (37.4%) |
| Reading glasses | 136 (57.9%) | 122 (51.9%) | 119 (50.6%) | 115 (48.9%) | 117 (49.8%) |
| Contact lenses | 2 (0.9%) | 3 (1.3%) | 3 (1.3%) | 2 (0.9%) | 2 (0.9%) |
| A magnifying glass | 3 (1.3%) | 5 (2.1%) | 4 (1.7%) | 4 (1.7%) | 6 (2.6%) |
| No preference | 22 (9.4%) | 17 (7.2%) | 17 (7.2%) | 8 (3.4%) | 12 (5.1%) |
| Missing/no response | 1 (0.4%) | 9 (3.8%) | 3 (1.3%) | 8 (3.4%) | 10 (4.3%) |
| Floor and ceiling effects were defined as >25% of participants endorsing the most severe health state (‘Very dissatisfied’ or ‘Always’) or least severe health state (‘Very satisfied’ or ‘Never’) for the NVS and NVCI respectively. *Floor and ceiling effects were not defined and assessed for the NVCP due to the non-ordered nature of response options. | | | | | |
