# Supplementary material for: Psychometric evaluation of the near activity visual questionnaire presbyopia (NAVQ-P) and additional patient-reported outcome items
Source: J Patient Rep Outcomes. 2024 Apr 9;8:41. doi: 10.1186/s41687-024-00717-9 (PMC11004101; doi:10.1186/s41687-024-00717-9)
Supplement: Supplementary file 15 — Supplementary Material 15 [file 41687_2024_717_MOESM15_ESM.rtf]

Inter-item correlation matrix for the NAVQ-P	
	Cross-sectional Analysis Population at Month 2 (N=227)	
NAVQ-P Item	Item 1. Reading Small Printed Text	Item 2. Reading on Smart-phone	Item 3. Reading on Tablet Device	Item 4. Reading on Laptop or Desktop	Item 5. Reading Labels on Receipts	Item 6. Reading Handwritten Text	Item 7. Seeing Keypad on a Digital Device	Item 8. Engaging in Hobbies	
Item 1. Reading Small Printed Text on Paper	-	 -	 -	 -	 -	 -	 -	 -	
Item 2. Reading on Smartphone	0.956	-	 -	 -	 -	 -	 -	 -	
Item 3. Reading on Tablet Device	0.927	0.973	-	 -	 -	 -	 -	 -	
Item 4. Reading on Laptop or Desktop	0.907	0.953	0.983	-	 -	 -	 -	 -	
Item 5. Reading Labels or Receipts	0.895	0.854	0.824	0.847	-	 -	 -	 -	
Item 6. Reading Handwritten Text	0.912	0.909	0.901	0.882	0.850	-	 -	 -	
Item 7. Seeing Keypad on a Digital Device	0.873	0.885	0.893	0.889	0.787	0.893	-	 -	
Item 8. Engaging in Hobbies	0.867	0.811	0.849	0.810	0.779	0.867	0.865	-	
Item 9. Seeing Fine Detail such as Sewing	0.902	0.894	0.920	0.907	0.884	0.864	0.848	0.883	
Item 10. Seeing things in Dim Light	0.901	0.843	0.814	0.821	0.858	0.868	0.744	0.780	
Item 11. Seeing things when Glare is Present	0.884	0.871	0.847	0.826	0.807	0.823	0.845	0.871	
Item 12. Seeing things in Bright Light	0.868	0.837	0.836	0.823	0.716	0.822	0.839	0.820	
Item 13. Reading text when Color is similar to background	0.904	0.858	0.869	0.854	0.808	0.843	0.832	0.849	
Item 14. Reading for Long Period of Time	0.841	0.807	0.787	0.817	0.774	0.804	0.796	0.813	
Item 15. Adjusting Vision from Long Distance to Short Distance	0.841	0.815	0.840	0.837	0.812	0.831	0.819	0.840	
Continued…	
NAVQ-P Item	Item 9. Seeing Fine Detail such as sewing	Item 10. Seeing things in Dim Light	Item 11. Seeing things when Glare is Present	Item 12. Seeing things in Bright Light	Item 13. Reading text when Color is Similar to the Background	Item 14. Reading for Long Period of Time	
Item 9. Seeing Fine Detail such as Sewing	-	 -	 -	 -	 -	 -	
Item 10. Seeing things in Dim Light	0.868	-	 -	 -	 -	 -	
Item 11. Seeing things when Glare is Present	0.864	0.836	-	 -	 -	 -	
Item 12. Seeing things in Bright Light	0.880	0.766	0.902	 -	 -	 -	
Item 13. Reading text when Color is Similar to the Background	0.868	0.868	0.866	0.892	 -	 -	
Item 14. Reading for Long Period of Time	0.812	0.777	0.836	0.853	0.826	-	
Item 15. Adjusting Vision from Long Distance to Short Distance	0.876	0.789	0.841	0.843	0.838	0.885	
Values represent polychoric correlation coefficients. Red shaded cells indicate correlations of r>0.90, which may suggest item redundancy. 	
