# Supplementary material for: Psychometric evaluation of the near activity visual questionnaire presbyopia (NAVQ-P) and additional patient-reported outcome items
Source: J Patient Rep Outcomes. 2024 Apr 9;8:41. doi: 10.1186/s41687-024-00717-9 (PMC11004101; doi:10.1186/s41687-024-00717-9)
Supplement: Supplementary file 16 — Supplementary Material 16 [file 41687_2024_717_MOESM16_ESM.docx]

| **NAVQ-P Rating Scale Model Item Parameters** | | | | | |
| --- | --- | --- | --- | --- | --- |
| **Items** | **a1** | **b1** | **b2** | **b3** | **c** |
| Item 1. Reading Small Printed Text on Paper | 1 | -4.73 | -1.4 | 1.82 | 0.000 |
| Item 2. Reading on Smartphone | 1 | -4.73 | -1.4 | 1.82 | -0.330 |
| Item 3. Reading on Tablet Device | 1 | -4.73 | -1.4 | 1.82 | -0.580 |
| Item 4. Reading on Laptop or Desktop | 1 | -4.73 | -1.4 | 1.82 | -0.708 |
| Item 5. Reading Labels or Receipts | 1 | -4.73 | -1.4 | 1.82 | 1.412 |
| Item 6. Reading Handwritten Text | 1 | -4.73 | -1.4 | 1.82 | -1.948 |
| Item 7. Seeing Keypad on a Digital Device | 1 | -4.73 | -1.4 | 1.82 | -1.913 |
| Item 8. Engaging in Hobbies | 1 | -4.73 | -1.4 | 1.82 | -1.319 |
| Item 9. Seeing Fine Detail such as Sewing | 1 | -4.73 | -1.4 | 1.82 | 0.942 |
| Item 10. Seeing things in Dim Light | 1 | -4.73 | -1.4 | 1.82 | 0.828 |
| Item 11. Seeing things when Glare is Present | 1 | -4.73 | -1.4 | 1.82 | -0.533 |
| Item 12. Seeing things in Bright Light | 1 | -4.73 | -1.4 | 1.82 | -1.697 |
| Item 13. Reading text when Color is similar to background | 1 | -4.73 | -1.4 | 1.82 | -0.043 |
| Item 14. Reading for Long Period of Time | 1 | -4.73 | -1.4 | 1.82 | 0.166 |
| Item 15. Adjusting Vision from Long Distance to Short Distance | 1 | -4.73 | -1.4 | 1.82 | -0.632 |
