# Supplementary material for: Psychometric evaluation of the near activity visual questionnaire presbyopia (NAVQ-P) and additional patient-reported outcome items
Source: J Patient Rep Outcomes. 2024 Apr 9;8:41. doi: 10.1186/s41687-024-00717-9 (PMC11004101; doi:10.1186/s41687-024-00717-9)
Supplement: Supplementary file 10 — Supplementary Material 10 [file 41687_2024_717_MOESM10_ESM.docx]

01 February 2024

Attn: Journal of Patient Reported Outcomes (JPRO)

Editorial Office

Dear Editor,

Thank you for providing feedback on the manuscript we recently submitted, titled ‘Psychometric evaluation of the Near Activity Visual Questionnaire Presbyopia (NAVQ-P) and additional patient-reported outcome items’. We appreciate the feedback provided by the reviewers and have considered each of the comments and provided detailed responses in Table 1. The manuscript has been revised in response to the reviewer’s comments. A tracked changes and a clean version are provided for your reference. We trust that we have been able to adequately address the comments raised by the reviewers to be suitable for publication and informative for readers.

We look forward to hearing from you on the outcome of the manuscript. If you have any questions, or wish to discuss anything, please do not hesitate to contact us.

Yours sincerely,
Corresponding Author

**Reviewer 1**

**Comment: A major weakness lies in the potential bias introduced by the study's population. The data used for psychometric validation were collected from a Phase IIb clinical trial with participants from the** **United States, Japan, Australia, and Canada. The lack of diversity in the study population may limit the generalizability of the findings to a more diverse global population, potentially affecting the external validity of the instruments. To enhance the robustness and applicability of the measures, future research should aim to include a more geographically and culturally diverse sample of individuals with presbyopia, ensuring that the psychometric properties established in this study are representative across a broader range of demographics and clinical settings.**

Response to comments: The authors thank the reviewer for reviewing the manuscript and providing this feedback.

We appreciate the point raised by this comment due to the importance of ensuring generalisability of the study findings to future samples. As the reviewer highlights, the population used in this psychometric study was limited to the eligibility criteria and sample recruited in the Phase IIb clinical trial. While we acknowledge it would be useful to include a more diverse range of countries in a validation study such as this, we do not believe this has compromised the findings of this study with regard to generalisability since the sample included a high proportion from culturally distinct countries (e.g. n=45 (19.8%) from Japan) and races (n=51 [22.5%] Asian).

However, since we appreciate this sample included participants from only four countries, we have added a statement to the discussion to note that a more geographically and culturally diverse sample of individuals and also including a more ‘real world’ sample should be considered in future.

Action: Statement added to discussion to note this as an area of improvement for future research (page 19, line 26-38).

**Reviewer 2**

**Comment: Major: While this is a reasonably interesting manuscript, I find that it has 2 significant methodological flaws which preclude me from recommending it for publication in JPRO: 1) the case for differences in the phakic presbyopia population vs those for whom the NAVQ-P was developed (the validation study) is not made strongly enough to motivate these analyses (and if indeed this is the primary rationale then a content/concept elicitation phase would have been more appropriate). In fact, despite the contention in the background section that the purpose of the study was to "to modify the NAVQ for use in people with phakic presbyopia", the first sentence of the discussion states that there is "evidence supporting the content validity of the NAVQ-P in phakic presbyopia" (although such evidence appears to be mis-cited in this manuscript as only 1 of the 6 references are about the NAVQ)**

Response to comments: The authors thank the reviewer for reviewing the manuscript and providing detailed feedback and comments on these various aspects.

**Re differences between pseudo-phakic and phakic presbyopia:**

Text has been added to the background detailing the differences between pseudo-phakic and phakic presbyopia to provide stronger evidence in support of the current research. Pseudo-phakic patients may have corneal scars and residual corneal irregularities from prior incisions. They are also typically older and as a result may experience increased prevalence of symptoms such as dry eyes. Because of these differences, symptoms and impacts relevant and important to assess in pseudo phakic populations may not be relevant to purely phakic populations. We would also like to point out that this was not the only justification for updating the original scale. As is mentioned in the background, for example, it was noted that some items in the original scale were outdated and did not reflect changes in technology which have occurred since the questionnaire was developed. Therefore, even if the reviewer may not agree with the importance of differences between phakic and pseudo phakic patients, we suggest that there is still adequate justification for the need to update the instrument, and the value of validating the new instrument.

**The purpose of the current study:**

Further text has been added to the background to clarify that the current study was the final stage in the rigorous research process to modify the NAVQ and to make clearer that a key driver was to ensure it reflected the use of digital screens in the modern world. The current study aimed to determine the scoring and psychometric properties and interpretability of the NAVQ-P and builds on earlier research which provided evidence to support the qualitative content validity of the modified instrument (detailed descriptions of this earlier research is provided in specific publications referenced in the background section). Thank you for highlighting that the evidence for content validity of the NAVQ-P has been mis-cited. The citations have now been updated to correct the error.

Action: Text added to the background to provide further evidence of the differences between the pseudo-phakic population for whom the original NAVQ was developed and the phakic population for whom the updated NAVQ-P in the current study was developed (page 4, line 30-45). Background also updated to better convey that a concept elicitation and cognitive debriefing phase of research did precede this psychometric validation study (page 4 line 45 to page 5 line 2).

Text added to the background to provide further details about the rigorous development process for the NAVQ-P, in line with best practice guidelines, including qualitative research to establish evidence for content validity (page 5, line 12-14).

**Comment: 2) the data come from an RCT testing an intervention to improve presbyopia, and analyses were undertaken across pooled treatment and placebo groups and at a 2-month follow-up timepoint. Therefore, psychometric results cannot be assumed to be uncontaminated by treatment effects.**

Response to comments: Thanks for this comment. The psychometric analyses were conducted at Month 2 in the cross-sectional analysis population since post-treatment timepoints were expected to have a greater distribution of responses compared to pre-treatment responses (as stated in Table 2 of the manuscript) leading to larger variability in scores. This was indeed the case as seen by the item response distributions (Supplementary File 4 and 5). We believe this approach enhanced the psychometric analysis which exploits variability in responses. We do not expect the psychometric properties of the instrument to be influenced by treatment effects, and the use of data from a sample pooled across treatment and placebo groups guards against this. We accept that the data being from an RCT should be fully acknowledged (which it has been), with this being the context of use, but disagree that this is a major limitation.

**Comment: Refine the study purpose to better reflect whether the purpose was restricted to evaluating, refining or validating the NAVQ-P and/or also to validate the additional single-items as stand-alone assessments or as adjuncts to the NAVQ-P.**

Response to comments: The purpose of this study was to validate the NAVQ-P as well as the additional single-item measures (NVCI, NVS and NVCP) which assess separate concepts to the NAVQ-P. This should now be clarified in the background through updates made in response to other reviewer’s comments.

Action: See background section (page 5 line 2-29).

**Comment: 227 participants completed all assessments at baseline and 4 follow-up time points (week 2, and months 1, 2, & 3). Distance-corrected near visual acuity (DCNVA) was also assessed at each of these timepoints. Analyses were based in the pooled data across treatment arms from the parent RCT. Overall, table 1 does a nice job of presenting the rationale for the analyses undertaken. However, given that, some of the information here could be augmented at a more conceptual level - for example, why do both a CFA and an IRT analysis? How do they complement each other and/or answer different questions?**

Response to comments: We agree with the reviewer that both CFA and IRT to some degree answer similar question (dimensionality) and are mathematically related (see e.g. McDonald, R. Test Theory: A Unified Treatment). However, each method has its own niche in looking at the measurement model underlying the data and therefore both methods can be seen as complementary. The Rasch model used in our IRT analysis has many specific properties (e.g. specific objectivity) that cannot be effectively captured within a CFA framework. Some differences between CFA and IRT are outlined below:

- CFA provides detailed information about global fit based on how well the proposed measurement model reproduces the observed covariance/correlation matrix.
- IRT provides an indication of item fit (infit, outfit) of each item to the Rasch measurement principles.
- Good fit to Rasch model provides solid basis to simple total score via specific objectivity property. Although this is to some degree possible also via CFA, using IRT framework is more straightforward.
- CFA provides more detailed information on partitioning of item variance (communality and uniqueness).
- CFA can capture and evaluate impact of local sources of misfit (e.g. correlated measurement errors) .

Overall, IRT is a useful method in examining individual item characteristics or establishing composite scores for respondents; and CFA is a useful method when assessing the structural makeup of a scale and whether a set of items can appropriately be used collectively to measure a construct. Considering both of these aforementioned points were primary aims within the study, it felt appropriate and robust to use both methods concurrently.

Action: Text added to ‘Statistical and data analysis methods’ section to provide a short justification (page 8, line 53-57).

**Comments: The cross-sectional analysis sample was assessed at 2-months when participants may have been experiencing significant treatment effects (relative to their own baseline and relative to the other treatment groups) if the treatment was effective, therefore including all patients in the analyses may have affected results. In particular test-retest reliability analysis seems misguided as the foundation of any test-retest reliability estimate is based in the assumption that the quantity of the underlying latent variable not changing. However, the manuscript fails to discuss the outcomes of the parent study and how the treatment arms may have impacted their own results and conclusions.**

Response to comments: The cross-sectional analysis sample was only used for analyses that were cross-sectional (single timepoint). As a result, changes due to treatment that some of those patients may have experienced between baseline and Month 2 are not relevant to the analyses. What is desirable for cross-sectional psychometric analyses is to include a sample which is distributed across the possible severity range (rather than, say only very mild or only very severe patients). As is highlighted in Table 1 in the manuscript, Month 2 was selected for the cross-sectional analyses because it was expected to have a greater distribution than would be present at Baseline, because the trial inclusion criteria required that all participants had to have moderate or severe presbyopia. Thus, we consider that the use of Month 2 data for the cross-sectional analyses was a strength, not a weakness of the study.

As detailed in Table 1, the test-retest analyses used the test-retest analysis population, rather than the cross-sectional analysis population. As explained in Table 1, the test-retest analysis population included only patients who were ‘stable’ between either Months 2 and 3 or between Week 2 and Month 1 as defined by the external anchors.

**Comments: The inclusion of the single-item measures is problematic. These items were developed by the authors for use in this study (it would appear), and therefore do not comprise adequate validation standards by which to judge the NAVQ. Specifically, a) 'known-groups' validity evidence should not be supported through examination of group-differences in these measures and in fact, the known-groups analyses conclusions are almost meaningless here as they are based entirely in a large 'known-groups' differences in the new single-item measures and no differences were found in actual objective acuity (DCNVA).**

Response to comments: Thank you for your comments regarding the validity of the single item measures. We assume the reviewer is referring to the single PGI items which were used as anchor measures in the current study. A previous publication provided evidence for the validity of the study instruments including the PGI items (Bentley et al 2021). All additional study measures (including the PGI items) were developed in parallel to the NAVQ-P and were subject to the same rigorous development process, following methodology for clinical outcome assessment development recommended by regulatory bodies including the FDA. However, we do accept the point that these items were developed specifically to support validation and meaningful change analyses as part of this study, which is a limitation. This limitation has now been more fully acknowledged in the discussion. Nevertheless, we suggest that their use to define known groups is still not without value, particularly given that DCNVA does not directly measure visual functioning. Hopefully the addition to the discussion addresses the concern adequately.

Action: A statement has been added to background section to clarify that the additional study measures were developed in parallel to the NAVQ-P and were subject to the same rigorous development process (page 5, line 12-14). Additionally, text has been added to the discussion to acknowledge the limitation of the PGI-S for known-groups validation and notes further study using other independently established measures of near vision functioning is warranted (page 21, line 2-12).

**Comments: The contention in table 2 that "The NVCI, NVS, and NVCP were not included in these (CFA) analyses as these are single-item measures for which CFA is not applicable" is not exactly appropriate as these newly developed single items may have fruitfully been included in the single-latent CFA model, this strategy could have imparted information about the single-items themselves as well as the fit and functioning of the NAVQ.**

Response to comments: Thank you for this comment and we agree this statement should be adjusted to make things clearer. We assume that the reviewer suggests that the additional single-item measures could have been included in the CFA as predictors and not as indicators of the latent construct (the latter would be meaningful only if there had been the expectation that these items would fit under the single latent factor which was not the case). These single items are intended to measure near vision correction independence (NVCI) and near vision satisfaction (NVS) and designed to be assessed as specific concepts completely distinct from near vision functioning assessed by the NAVQ-P. Adding these single-item measures as predictors would introduce a MIMIC model which could potentially answer interesting questions regarding their relationships to the concept of near vision functioning and provide useful information about convergent and divergent validity. However, such relationships are provided in Table 6 and thus the inclusion of these items in CFA would be somewhat repetitive. We’ve adjusted the text in the methods table to try to make this clearer. Thus, we accept that inclusion in the CFA may have imparted information about how the single-item measures relate to near vision functioning and the latent structure(s) assessed by the NAVQ-P but we did not consider this beneficial to run such analysis within CFA and instead present this information in the convergent validity section.

Actions: Adjusted the text in the methods table (Table 2).

**Comments: The difference between a 'constrained' and an 'unconstrained' model is not described. The CFA tested a unidimensional latent structure so the reader is left wondering what parameters were constrained and why, and more importantly what research question was being asked with this approach? Constrained and unconstrained model comparisons are more commonly used in measurement invariance studies where research questions about parameter differences across populations are of interest. The authors are recommended to provide a reference for this analysis and to more clearly specify why it was done.**

Response to comments: Thank you for your comment. The comparison of constrained vs unconstrained CFA models were employed to assess whether a weighted or unweighted daily sum score was needed by checking homogeneity of factor loadings. This was evaluated empirically – we compared fit of the model where factor loadings were estimated freely, with the model where factor loadings were constrained to be the same. In the case that factor loadings can be considered equal (i.e. model with constrained factor loadings will not fit significantly worse compared to model with freely estimated factor loadings) across items, an unweighted sum score was proposed. For models with heterogeneous factor loadings, weighted sum score is more appropriate. We appreciate that it may, to some extent, have a contextual overlap with aims of Rasch modelling results, but we see this as a useful additional analysis for establishing composite score.

We have included a small description of constrained vs unconstrained in Table 2 , with further clarifying information and a reference.

Action: A statement added to Table 2 along with further clarifying information and reference added.

**Comments: Conclusions**

**A revision to the manuscript that ties analyses and results to the outcomes of the parent study may be in a position to make a contribution to the literature (since review of the results on clinicaltrials.gov suggests no effects). Such a revision should be pared down to include fewer analyses that are more strongly motivated in terms of answering specific questions about the NAVQ (and also potentially the utility of the single-item measures). Given that ultimately this study resulted in omission of 1 item and that the omission was motivated not only by psychometric analysis but also through consultation with a clinician, it does appear that a content/elicitation phase may have been useful and perhaps sufficient.**

Response to comments: We take on board the reviewers point that it is arguably appropriate to acknowledge the fact that the trial the data was taken from was unsuccessful, with no evidence of treatment differences. A statement around this has been added to the discussion. However, we suggest that the fact that the psychometric analyses are based on data pooled across treatment groups, and with change groups defined using anchors, means that the psychometric data are still appropriate to report. Therefore, we don’t agree with substantially paring back the analyses. All of the analyses documented provide insight into the psychometric performance of the NAVQ-P, and as such we suggest they are relevant to present.

The background section has also been updated to better convey that a concept elicitation and cognitive debriefing phase of research did precede this psychometric validation study.

Action: A statement has been added to the penultimate paragraph of the discussion to refer to the results of the parent study (page 21 line 48 to page 22 line 7).

The background has been updated to better convey that a concept elicitation and cognitive debriefing phase of research did precede this psychometric validation study (page 4 line 45 to page 5 line 2).
